# Resolving the identification of weak‐flying insects during flight: a coupling between rigorous data processing and biology

**DOI:** 10.1111/afe.12453

**Published:** 2021-06-02

**Authors:** Kirsty L. Hassall, Alex Dye, Ilyas Potamitis, James R. Bell

**Affiliations:** ^1^ Computational and Analytical Sciences Rothamsted Research West Common, Harpenden AL5 2JQ U.K.; ^2^ Rothamsted Insect Survey Rothamsted Research West Common, Harpenden AL5 2JQ U.K.; ^3^ Department of Music Technology and Acoustics Engineering Hellenic Mediterranean University Crete Greece

**Keywords:** Aphid, beetle, insect classification, random forest classification, wingbeat harmonics

## Abstract

1. Bioacoustic methods play an increasingly important role for the detection of insects in a range of surveillance and monitoring programmes.
2. Weak‐flying insects evade detection because they do not yield sufficient audio information to capture wingbeat and harmonic frequencies. These inaudible insects often pose a significant threat to food security as pests of key agricultural crops worldwide.
3. Automatic detection of such insects is crucial to the future of crop protection by providing critical information to assess the risk to a crop and the need for preventative measures.
4. We describe an experimental set‐up designed to derive audio recordings from a range of weak‐flying aphids and beetles using an LED array.
5. A rigorous data processing pipeline was developed to extract meaningful features, linked to morphological characteristics, from the audio and harmonic series for six aphid and two beetle species.
6. An ensemble of over 50 bioacoustic parameters was used to achieve species discrimination with a success rate of 80%. The inclusion of the dominant and fundamental frequencies improved prediction between beetles and aphids because of large differences in wingbeat frequencies.
7. At the species level, error rates were minimized when harmonic features were supplemented by features indicative of differences in species' flight energies.

## Graphical abstract

Data processing pipeline for the automatic identifcation of weak‐flying insects. Insects fly through an opto‐acoustic sensor triggering an audio recording to be made of the sound of their flight. Data are then processed to enable feature extraction. These features are fed into classification algorithms, namely random forests, which are then checked for validity before being sent to the end user for interpretation.

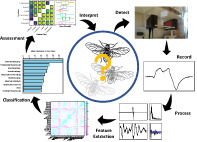

**Abbreviations**
AAU: arbitrary amplitude units
ANOVA: analysis of variance
clErr: class error
DSS: decision support system
GAM: generalized additive model
IQR: interquartile range
LED: light‐emitting diode
RMS: root mean square
SD: secure digital
TNR: true negative rate
TPR: true positive rate
wAcc: weighted accuracy

## Introduction

Over the last 30 years, agriculture has been in the midst of a digital revolution with the increasing availability of sensor technology and associated collection of ‘big data’ aiming at improving the sustainability of food production systems. These technologies exist at all levels of the agricultural system, from defining management zones from yield monitor data (Milne *et al*., 2012), drones and augmented reality (Huuskonen & Oksanen, 2018) to pest detection using UAVs (Tetila *et al*., 2020). Many of these technologies feed into decision support systems (DSS) further enabling the implementation of precision agriculture (Cancela *et al*., 2019; Zhai *et al*., 2020). Despite this, insect pests remain a key challenge. Automatic insect pest detection is a long sought‐after goal that began in the 1950s and has still yet to reach maturity (Lowe & Dromgoole, 1958; Cardim Ferreira Lima *et al*., 2020). For many years, image recognition systems have been at the forefront but increasingly, bioacoustic methods are playing an important role for the detection of insects in a diverse range of surveillance and monitoring programmes. Almost exclusively, these programs use sound recordings to detect species and groups across the audible range of human hearing (Chen *et al*., 2014). Typically, model organisms have included mosquitoes, fruit flies, hawkmoths and crickets using a simple microphone set‐up (Montealegre‐Z *et al*., 2011; Potamitis & Rigakis, 2016b; Mukundarajan *et al*., 2017). Although the songs of some cicadas can generate in excess of 100 dB of sound pressure (Sanborn & Phillips, 1995), there is an important group of small insects that are effectively silent in flight, which pose a much more significant threat. For example, the peach potato aphid, *Myzus persicae* (Sulzer, 1776) (Hemiptera: Aphididae), compromises worldwide food security through the transmission of 100 different plant viruses, and is consequently one of the world's top 10 pests (Willis, 2017; CABI, 2020). Yet, these small insects, no more than a couple of millimetres long and of the upmost agricultural importance, are not the focal interest of bioacoustics monitoring, despite their profound and lasting impact on food quality and quantity.

Aphid flights are so weak that they fall well below the lowest human hearing range of 40 dB at 100 Hz and are effectively silent, producing a wingbeat frequency that is nearly an order of magnitude weaker than some mosquitoes (Byrne *et al*., 1988; Smith, 1999; Moore & Miller, 2002; Potamitis & Rigakis, 2016b; Tercel *et al*., 2018). Further, the rate of progress in flight is very weak, and no more than 0.70 m/s^−1^ under laboratory conditions for some well‐studied aphids (Thomas *et al*., 1977).

Recently, opto‐acoustic methods have provided a novel way of capturing the flight of these small insects using phototransistors and infrared light (Ouyang *et al*., 2015; Potamitis & Rigakis, 2016a,b). Here, the use of both the extinction of light and backscattered light principles has been shown to perform better than audio (Potamitis *et al*., 2015; Potamitis & Rigakis, 2016b).

In parallel to the evolution of sensor technologies has been a rapid development in computational techniques. Classification problems once thought to be impossible can be tackled by any number of different data science techniques. Such techniques have been widely applied to the classification of species from audio measurements and, in general, the literature approaches this problem in two ways; the first approach generates a ‘dictionary’ of features for each species through unsupervised learning methods such as clustering and nearest neighbour classification. A new unknown species is then allocated to the closest ‘word’. Such methods have been shown to perform relatively poorly (Moore & Miller, 2002; Potamitis, 2014; Potamitis *et al*., 2015). The second, more successful, approach focuses on species classification through supervised learning algorithms that learn from labelled data. These include artificial neural networks (Moore, 1991; Moore & Miller, 2002), Gaussian mixture models (Potamitis, 2014; Ouyang *et al*., 2015), random forests, support vector machines and gradient boosting classifiers (Potamitis *et al*., 2015). Deep learning approaches are seeing an exponential increase in their usage but as highlighted in the studies by Chen *et al*. (2014) and Kiskin *et al*. (2020), such methods also require formidable sample sizes. Conversely, convolutional neural networks have been used to good effect in data scarce scenarios (Kiskin *et al*., 2020).

Not only do classification methods vary in the chosen algorithm but also in the choice of input data. Chen *et al*. (2014) attribute the stagnation of insect classification in part to the overreliance on a single feature of wingbeat frequency; often the fundamental frequency or the rate of wing flap. This was observed more than 20 years previously (Moore, 1991) where error rates in classification of mosquitoes increased by 33% when only using the wingbeat frequency, compared with using the entire frequency spectra. Later, however, it was found that using the first 17 harmonics was as effective as using the entire frequency spectra to classify five different aphid species (Moore & Miller, 2002). Similarly, 12 features from the cepstrum have been used as input to the Gaussian mixture models for classifying mosquitoes (Ouyang *et al*., 2015). This contrasts with the study by Potamitis *et al*. (2015)) and references therein who argue that the unprocessed spectra are a better choice than more sophisticated features coming from individual harmonics. This is taken further in other studies (Chen *et al*., 2014) where the frequency spectra are supplemented with additional covariates, such as time of flight and where prior information on insect behaviour is available for inclusion in the Bayes classifier. A less common approach is to move away from the frequency domain and to instead use wavelet transformations of the audio (Kiskin *et al*., 2020), which arguably loses biological interpretability in relation to insect flight.

The literature shows little consensus on the convergence of a single approach but individual studies highlight nuances in specific application areas. It is not known whether the performance of algorithms and associated processing of data differs due to targeted species, experimental conditions, tuning parameters, or most likely a combination of all three. However, the choice of both algorithm and data processing should be made in the context of why discrimination is needed. In this paper, we aim to couple together successful classification along with biological insight and as such, consider both rigorous data processing to extract morphologically meaningful parameters and machine learning algorithms to develop classification models. Furthermore, we aim to show that an ensemble of bioacoustic parameters and indices can be used to distinguish between groups and species of the agriculturally and economically important, but often overlooked, weak‐flying insects.

## Materials and methods

### Flight experiments

Opto‐acoustic recorders capture the variation of light when an insect passes through a light beam. Both the main body and the wings cast a shadow in the emitter's light beam, known as the extinction of light principle, and this shadow is subsequently detected by a receiver photodiode array (Potamitis & Rigakis, 2016a). The Wingbeat Recorder® (Insectronics, Chania, Crete, Greece) was set up on a work surface in a laboratory, with white dividers either side of the set‐up preventing other equipment, lasers and light to interfere with the recording. During experimental conditions, the sensor was placed underneath a 15 000 mL heavy‐walled glass beaker (Duran™), which provided sufficient space for insects to behave as normal.

For each species studied, insects were either collected from the field or from insectary reared cultures and placed within the up‐turned beaker in which the sensor was enclosed (Fig. S1). Over a period of 2 days, insects were free to disperse in and around the sensor. Flights were automatically triggered when an insect entered the field of view of the LED array, generating a recording lasting 0.6 s. All flights were saved as an audio file on an SD card within the sensor along with average temperature and humidity covariates. Approximately 30 insects were used per experiment run, generating on average 50 recordings (ranging from 0 to more than 500) per run.

### Audio pre‐processing and feature extraction

**Figure 1:**
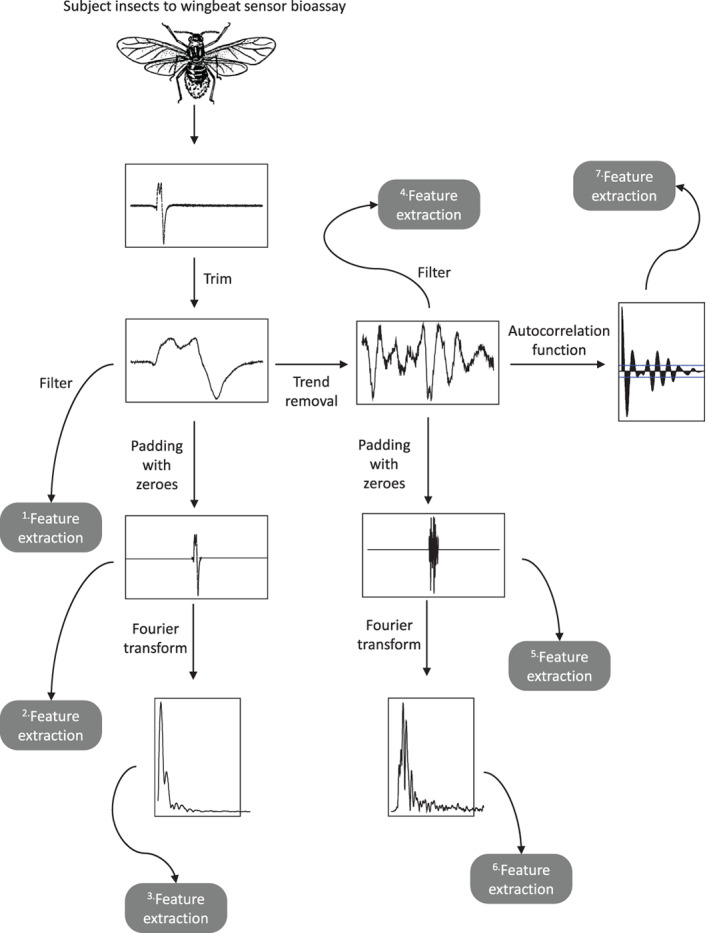
Data processing pipeline, indicating where features are extracted. Aphid illustration released under the creative commons Licence https://commons.wikimedia.org/wiki/File:Aphid_(PSF).Png.

A depiction of the audio processing steps is shown in Fig. 1. Amplitude of the audio (volume) was scaled according to the bit rate, *b*, (divided by 0.5 × 2^b^) to be expressed in arbitrary amplitude units (AAU). This allows a direct comparison between the amplitude across recordings sampled with different bit rates. Audio recordings were trimmed to remove the ‘silence’ at either end of the recording. A threshold of 0.0061 AAU was identified at which sound can be considered background noise. However, background noise exhibits stochasticity, and the first sound above this threshold does not necessarily indicate it is an insect flight. Thus, to determine the first and last true sound and therefore flight, any index for which the sound was above the threshold and identified as an outlier (on the temporal scale), defined as more than three times the interquartile range (IQR) away from the upper or lower quartiles, was considered stochastic variation above the threshold and not true sound (Fig. S2). Trimmed audio recordings consisting of fewer than 128 time points (a total of 0.01 s) were removed.

A filtering to remove background variation through a short‐time Fourier transform with Hanning window was applied to the trimmed audio recordings. Summary statistics (maximum amplitude, amplitude range, amplitude interquartile range, see Table 1) and measures of energy (crest factor, energy, power and root mean square (RMS), see Table 1) were obtained from the filtered trimmed audio (Feature extraction box 1 in Fig. 1). Additional summary statistics (amplitude index and temporal entropy, see Table 1) were obtained from zero padded trimmed audio (Feature extraction box 2 in Fig. 1).

**Table 1:** A summary of the features extracted from the audio recordings along with their definition

Extraction stage	Feature	Transformation	Feature type	Mathematical description	Description	Biological category
1	Maximum amplitude	Logarithm	Audio	$\max_{t}\left|a\left(t\right)\right|$	Maximum amplitude of a half cycle wingbeat centred at 0.	Flight energy
1	Amplitude range	Logarithm	Audio	$\max_{t}\;a\left(t\right)-\min_{t}\;a\left(t\right)$	The difference between the maximum peak and the minimum trough of a wingbeat recording.	Flight energy
1	Amplitude IQR	Logarithm	Audio	*q*_3_(*a*(*t*)) − *q*_1_(*a*(*t*)), where *q*_1_ and *q*_3_ denote the first and third quartile.	The difference between recorded amplitude at the third and first quartiles. A dampened measure of flight energy ignoring extreme values.	Flight energy
1	Power	Logarithm	Audio	$\frac{1}{n}\sum_{t}{\left[a\left(t\right)\right]}^{2}$, where *n* is the number of time points in the recording.	The average squared amplitude.	Flight energy
1	Root mean square (RMS)	Logarithm	Audio	$\sqrt{\frac{1}{n}\sum_{t}{\left[a\left(t\right)\right]}^{2}}$	The average squared amplitude, downweighted by a square root transformation, dampening influence of loud sounds.	Flight energy
1	Crest factor	Logarithm	Audio	$\max_{t}\left|a\left(t\right)\right|/\sqrt{\frac{1}{n}\sum_{t}{\left[a\left(t\right)\right]}^{2}}$	The ratio between the peak value relative to the RMS of the wingbeat cycle in the series.	Speed of wing transition relative to overall flight energy
2	Amplitude index	Logarithm	Audio	*q*_2_(*a* ^*^(*t*)), where *q*_2_ denotes the median.	The median value of the amplitude over time.	Flight energy
2	Temporal entropy		Audio	$\frac{-1}{\log n}\sum_{t}\;a^{*}\left(t\right)\log a^{*}\left(t\right)$	A function of Shannon evenness, the index estimates the variability in amplitude (loudness) over time.	Consistency of flight energy
3	Bioacoustics index (1)		Frequency	$\sum_{\xi =0}^{1000}\;\bar{f}\left(\xi \right)$	The area under the curve of the frequency spectra between 0–1000 Hz. This may include lower frequency body oscillations of the insect between 0–50 Hz.	Harmonic information including body oscillations
3	Bioacoustics index (2)		Frequency	$\sum_{\xi =50}^{1000}\;\bar{f}\left(\xi \right)$	The area under the curve of the frequency spectra between 0–1000 Hz. This will exclude lower frequency body oscillations of the insect between 0–50 Hz.	Harmonic information excluding body oscillations
3	Bioacoustics index (3)		Frequency	$\sum_{\xi =50}^{300}\;\bar{f}\left(\xi \right)$	The area under the curve of the frequency spectra between 50–300 Hz. This will only include low order frequencies including the fundamental frequency.	Harmonic information at low frequencies
3	Bioacoustics index (4)		Frequency	$\sum_{\xi =200}^{3000}\;\bar{f}\left(\xi \right)$	The area under the curve of the frequency spectra between 200–3000 Hz. This will only include higher‐order frequencies.	Harmonic information at high frequencies
3	Spectral entropy		Frequency	$\frac{-1}{\log n}\sum_{\xi }\bar{f}\left(\xi \right)\log\bar{f}\left(\xi \right)$	A function of Shannon evenness, the index estimates the variability in the frequency spectrum.	Smoothness of the harmonic series
3	Acoustic entropy		Audio‐frequency	$\frac{-1}{\log n}\sum_{t}\;a^{*}\left(t\right)\log a^{*}\left(t\right)\times \frac{-1}{\log n}\sum_{\xi }\;\bar{f}\left(\xi \right)\log\bar{f}\left(\xi \right)$	An index lying between 0 and 1, with 0 indicating a pure tone and 1 indicating random noise. (Sueur *et al*., 2008b).	Clarity of the recorded sound
3	Dominant frequency	Square root	Frequency	$f^{-1}\left(\max_{\xi >50}\;f\left(\xi \right)\right)$	The ‘loudest’ frequency, *i.e*. the frequency corresponding to the largest changes in amplitude such that the frequency exceeds body oscillations of at least 50 Hz.	(inverse) length of the largest oscillations
3	First harmonic		Harmonics	$h_{1}=f^{-1}\left(\max_{\xi }\;f\left(\xi \right)\right)$	The ‘loudest’ frequency, *i.e*. the frequency corresponding to the largest changes in amplitude. This will coincide with the dominant frequency where the largest frequency is over 50 Hz.	(inverse) length of the largest oscillations
3	Second harmonic		Harmonics	$h_{2}=f^{-1}\left(\max_{\xi ,\xi \neq h_{1}}\;f\left(\xi \right)\right)$	The second to tenth loudest frequency. Higher harmonics may be multiples of lower harmonics, where a repeating oscillation is detected.	(inverse) length of the higher order oscillations.
3	Third harmonic		Harmonics	$h_{3}=f^{-1}\left(\max_{\xi ,\xi \neq h_{1},h_{2}}f\left(\xi \right)\right)$		
3	Fourth harmonic		Harmonics	$h_{4}=f^{-1}\left(\max_{\xi ,\xi \neq h_{1},h_{2},h_{3}}f\left(\xi \right)\right)$		
3	Fifth harmonic		Harmonics	$h_{5}=f^{-1}\left(\max_{\xi ,\xi \neq h_{1}\ldots h_{4}}f\left(\xi \right)\right)$		
3	Sixth harmonic		Harmonics	$h_{6}=f^{-1}\left(\max_{\xi ,\xi \neq h_{1}\ldots h_{5}}f\left(\xi \right)\right)$		
3	Seventh harmonic		Harmonics	$h_{7}=f^{-1}\left(\max_{\xi ,\xi \neq h_{1}\ldots h_{6}}f\left(\xi \right)\right)$		
3	Eighth harmonic		Harmonics	$h_{8}=f^{-1}\left(\max_{\xi ,\xi \neq h_{1}\ldots h_{7}}f\left(\xi \right)\right)$		
3	Ninth harmonic		Harmonics	$h_{9}=f^{-1}\left(\max_{\xi ,\xi \neq h_{1}\ldots h_{8}}f\left(\xi \right)\right)$		
3	10th harmonic		Harmonics	$h_{10}=f^{-1}\left(\max_{\xi ,\xi \neq h_{1}\ldots h_{9}}f\left(\xi \right)\right)$		
4	GAM amplitude range	Logarithm	Audio	$\max_{t}\;g\left(t\right)-\min_{t}\;g\left(t\right)$	The difference between the peak and the trough of a GAM smoothed signal.	Flight behaviour
4	Maximum amplitude (g)	Logarithm	Audio	$\max_{t}\left|a\left(t\right)\right|$	As above but applied to the detrended audio signal	As above but applied to the detrended audio signal
4	Amplitude range (g)	Logarithm	Audio	$\max_{t}\;a\left(t\right)-\min_{t}\;a\left(t\right)$		
4	Amplitude IQR (g)	Logarithm	Audio	*q*_3_(*a*(*t*)) − *q*_1_(*a*(*t*)), where *q*_1_ and *q*_3_ denote the first and third quartile.		
4	Power (g)	Logarithm	Audio	$\frac{1}{n}\sum_{t}{\left[a\left(t\right)\right]}^{2}$, where *n* is the number of time points in the recording.		
4	RMS (g)	Logarithm	Audio	$\sqrt{\frac{1}{n}\sum_{t}{\left[a\left(t\right)\right]}^{2}}$		
4	Crest factor (g)	Logarithm	Audio	$\max_{t}\left|a\left(t\right)\right|/\sqrt{\frac{1}{n}\sum_{t}{\left[a\left(t\right)\right]}^{2}}$		
5	Amplitude index (g)	Logarithm	Audio	$q_{2}\left(\;{\tilde{a}}^{*}\;\left(t\right)\right)$		
5	Temporal entropy (g)		Audio	$\frac{-1}{\log n}\sum_{t}\;{\tilde{a}}^{*}\left(t\right)\log\;{\tilde{a}}^{*}\left(t\right)$		
6	Bioacoustics index (1) (g)		Frequency	$\sum_{\xi =0}^{1000}\;\bar{\tilde{f}}\left(\xi \right)$		
6	Bioacoustics index (2) (g)		Frequency	$\sum_{\xi =50}^{1000}\;\bar{\tilde{f}}\left(\xi \right)$		
6	Bioacoustics index (3) (g)		Frequency	$\sum_{\xi =50}^{300}\;\bar{\tilde{f}}\left(\xi \right)$		
6	Bioacoustics index (4) (g)		Frequency	$\sum_{\xi =200}^{3000}\;\bar{\tilde{f}}\left(\xi \right)$		
6	Spectral entropy (g)		Frequency	$\frac{-1}{\log n}\sum_{\xi }\;\bar{\tilde{f}}\left(\xi \right)\log\;\bar{\tilde{f}}\left(\xi \right)$		
6	Acoustic entropy (g)		Audio‐frequency	$\frac{-1}{\log n}\sum_{t}\;{\tilde{a}}^{*}\left(t\right)\log\;{\tilde{a}}^{*}\left(t\right)\times \frac{-1}{\log n}\sum_{\xi }\;\bar{\tilde{f}}\left(\xi \right)\log\;\bar{\tilde{f}}\left(\xi \right)$		
6	Dominant frequency (g)	Square root	Frequency	${\tilde{f}}^{-1}\left(\max_{\xi >50}\tilde{f}\left(\xi \right)\right)$		
6	First harmonic (g)		Harmonics	${\tilde{h}}_{1}={\tilde{f}}^{-1}\left(\max_{\xi }\tilde{f}\left(\xi \right)\right)$		
6	Second harmonic (g)		Harmonics	${\tilde{h}}_{2}={\tilde{f}}^{-1}\left(\max_{\xi ,\xi \neq {\tilde{h}}_{1}}\tilde{f}\left(\xi \right)\right)$		
6	Third harmonic (g)		Harmonics	${\tilde{h}}_{3}={\tilde{f}}^{-1}\left(\max_{\xi ,\xi \neq {\tilde{h}}_{1},{\tilde{h}}_{2}}\tilde{f}\left(\xi \right)\right)$		
6	Fourth harmonic (g)		Harmonics	${\tilde{h}}_{4}={\tilde{f}}^{-1}\left(\max_{\xi ,\xi \neq {\tilde{h}}_{1},{\tilde{h}}_{2},{\tilde{h}}_{3}}\tilde{f}\left(\xi \right)\right)$		
6	Fifth harmonic (g)		Harmonics	${\tilde{h}}_{5}={\tilde{f}}^{-1}\left(\max_{\xi ,\xi \neq {\tilde{h}}_{1}\ldots {\tilde{h}}_{4}}\tilde{f}\left(\xi \right)\right)$		
6	Sixth harmonic (g)		Harmonics	${\tilde{h}}_{6}={\tilde{f}}^{-1}\left(\max_{\xi ,\xi \neq {\tilde{h}}_{1}\ldots {\tilde{h}}_{5}}\tilde{f}\left(\xi \right)\right)$		
6	Seventh harmonic (g)		Harmonics	${\tilde{h}}_{7}={\tilde{f}}^{-1}\left(\max_{\xi ,\xi \neq {\tilde{h}}_{1}\ldots {\tilde{h}}_{6}}\tilde{f}\left(\xi \right)\right)$		
6	Eighth harmonic (g)		Harmonics	${\tilde{h}}_{8}={\tilde{f}}^{-1}\left(\max_{\xi ,\xi \neq {\tilde{h}}_{1}\ldots {\tilde{h}}_{7}}\tilde{f}\left(\xi \right)\right)$		
6	Ninth harmonic (g)		Harmonics	${\tilde{h}}_{9}={\tilde{f}}^{-1}\left(\max_{\xi ,\xi \neq {\tilde{h}}_{1}\ldots {\tilde{h}}_{8}}\tilde{f}\left(\xi \right)\right)$		
6	10th harmonic (g)		Harmonics	${\tilde{h}}_{10}={\tilde{f}}^{-1}\left(\max_{\xi ,\xi \neq {\tilde{h}}_{1}\ldots {\tilde{h}}_{9}}\tilde{f}\left(\xi \right)\right)$		
7	Fundamental frequency (g)	Logarithm	Harmonics	1/*T*, where *T* is the period of the autocorrelation function.	A repeating and consistent frequency that relates to the wingbeat frequency (*i.e*. number of wingflaps per sec)	Wingbeat frequency

Let a(t) denote the amplitude of the trimmed audio at time t and a*(t) denote the Hilbert amplitude envelope at time t (22). Let f(ξ) denote the spectrum of a(t) at frequency ξ Hz. Let $\bar{f}\left(\xi \right)$ denote the mean spectrum. Let g(t) denote the GAM estimate of the long‐term trend and ã and $\tilde{f}$ be the amplitude and frequency of the GAM adjusted audio. Transformations are applied to the formulae listed. All log transformations are the natural logarithm. (g) Indicates feature has been calculated after removal of the long‐term trend. Indicated in the table is the stage at which the feature was extracted, corresponding to the labelled boxes in Fig. 1. The final column provides a description of the biological interpretation of each feature.

The frequency spectrum was calculated with a window length of 128 time points using a short‐time Fourier transform and applied to the trimmed audio, padded with zeroes to make a total recording of length 8192 (2^13^) time points. The dominant frequency was identified as the largest harmonic above 0.05 kHz. Harmonics were extracted in order of frequency peak height and the top 10, with no lower frequency limit, were recorded. Spectral summaries (bioacoustic indices at four different frequency ranges: 0–1000, 50–1000, 50–300, 200–3000 Hz; spectral entropy and the acoustic entropy, see Table 1) were calculated (Feature extraction box 3 in Fig. 1).

A long‐term trend, often consisting of a single peak and trough, was evident in the majority of recordings. It is thought that this long‐term trend relates to insect flight movement (*e.g*. a banking behaviour) rather than to wingbeat frequencies alone. As such, the frequency spectra extracted as above may have limited interpretability of the resulting harmonics, which, in particular, prohibits the estimation of the fundamental frequency. Thus, a second set of features are calculated after removal of this long‐term trend through smoothing, estimated *via* a generalized additive model (GAM). The GAM was fitting using thin plate regression splines and a maximum basis dimension of 1/50th of the length of the trimmed audio or of dimension 10, whichever was bigger. Summary statistics (maximum amplitude, amplitude range and amplitude interquartile range, see Table 1) and measures of energy (crest factor, energy, power and root mean square (RMS), see Table 1) and also the maximum amplitude of the estimated GAM were obtained from the filtered trimmed audio (Feature extraction box 4 in Fig. 1). Additional summary statistics (amplitude index and temporal entropy) were obtained from zero padded trimmed audio (Feature extraction box 5 in Fig. 1). Harmonic features (dominant frequency, top 10 harmonic peaks, bioacoustic indices at four different frequency ranges: 0–1000, 50–1000, 50–300, 200–3000 Hz; spectral entropy and the acoustic entropy) were extracted from the frequency spectrum calculated on the zero‐padded, trend‐removed, trimmed audio (Feature extraction box 6 in Fig. 1).

To calculate the fundamental frequency, peaks were identified in the modulus of the autocorrelation function of the detrended audio, ensuring peaks were no closer than 10 time points (0.001 s) apart. The fundamental frequency was then calculated as the inverse of the time of the first peak (Feature extraction box 7 in Fig. 1).

Audio processing was done in the statistical software package, R, using packages seewave (Sueur *et al*., 2008a) for the Fourier transform, calculation of the harmonics and the calculation of temporal, spectral and acoustic entropies. The package soundecology (Villanueva‐Rivera & Pijanowski, 2018) was used to calculate the bioacoustics index, and the mgcv package (Wood, 2011) was used for the GAM estimation.

### Data

A total of 5026 audio recordings were available. Ninety‐eight of these did not exceed the required minimum audio length of 0.016 s, potentially because individuals did not fly through the whole sensor or were flying vertically through the sensor, and no features were extracted. Of the remaining 4928 observations, for which up to 52 features (as listed in Table 1) were calculated, 70% were randomly allocated to the training set and 30% to the validation set. Four aphid species (*Aphis fabae*, *Sitobion avenae*, *Myzus persicae* and *Rhopalosiphum padi*) were studied because they are global pests and reported on weekly by the Rothamsted Insect Survey (RIS) to growers (https://insectsurvey.com/aphid‐bulletin). *Drepanosiphum platanoidis* and *Periphyllus testudinaceus* are two additional aphid species that are included in our analyses and while neither is a crop pest, they are likely to be sampled by a sensor deployed in the field, particularly near sycamores and maples close to field margins. *Psylliodes chrysocephala* and *Brassicogethes aeneus* pose a serious threat to oilseed rape (*Brassica napus*) and other brassicas and are featured weekly in RIS' non‐aphid commentary (https://insectsurvey.com/ris‐remarks). Due to the small number of recordings for *M. persicae* and *R. padi*, these species were excluded from the random forest analysis, but are included in the basic analyses of flight. The number of observations for each species within each dataset is given in Table 2.

**Table 2:** Number of audio recordings with feature information listed by species in the complete, training and validation datasets

	Species					
Insect order	Common	Latin	Number of observations	Training	Validation	Wing
Hemiptera	Sycamore aphid	*Drepanosiphum platanoidis*	3323	2351	972	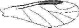
Hemiptera	English grain aphid	*Sitobion avenae*	274	193	81	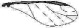
Hemiptera	Maple aphid	*Periphyllus testudinaceus*	113	76	37	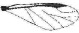
Hemiptera	Black bean aphid	*Aphis fabae*	161	120	41	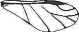
Hemiptera	Peach‐potato aphid	*Myzus persicae*	15			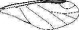
Hemiptera	Bird cherry‐oat aphid	*Rhopalosiphum padi*	8			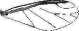
Coleoptera	Pollen beetle	*Brassicogethes aeneus*	848	566	282	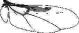
Coleoptera	Cabbage stem flea beetle	*Psylliodes chrysocephala*	186	127	59	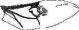
	Total		4928	3433	1472	

Illustration of wing venation for each species.

A total of 52 features were calculated for each audio recording. Seven different feature sets were considered and are shown in Table S1. The first set considers all 52 features. The second considers the 25 features calculated without detrending, whereas the third feature set considers the 27 features calculated on the detrended audio. Feature set 4 considers only the frequencies of the harmonic peaks, calculated both before and after signal detrending. Feature set 5 extends set 4 to also include the frequency indices such as the bioacoustics index, spectral entropy and dominant and fundamental frequencies. Feature sets 6, 7 and 8 consist of the representative features from a hierarchical cluster analysis with complete linkage on the correlation matrix of standardized features with 3, 5 and 14 clusters (Fig. S3). Representative features were defined to be the feature closest to the cluster centroid.

### Statistical analysis

A linear model was fitted to each feature including covariates; humidity and temperature and an explanatory variable indicative of species. A Type II ANOVA table was produced showing the effect of dropping each term while retaining all others in the model. Where necessary, variables were transformed to ensure homogeneity of variance as listed in Table 1.

Random forests (Breiman, 2001) were used to classify observations. Given the high levels of data imbalance across species, balanced random forests were implemented. Balanced random forests resample the data according to a set of defined class‐specific sample sizes. Considerable tuning of these class sample sizes is required, and our criteria for tuning were to balance the class‐specific error rates. The chosen set of sample sizes for *A. fabae*, *P. chrysocephala*, *S. avenae*, *P. testudinaceus*, *B. aeneus*, *D. platanoidis* were 75, 75, 120, 50, 120, 300 for datasets including observations with missing values and 60, 60, 96, 40, 96, 240 for datasets excluding observations with missing values, respectively. Hyper‐parameters were tuned through an assessment of both the out‐of‐bag error rate and predictive accuracy. Selected hyper‐parameters were to grow 1000 trees trying 10 randomly selected variables at each split. Missing values were handled through the inbuilt option na.roughfix, which imputes missing values by the variable median. To compute the accuracy on validation data, missing values were replaced by the median of each feature as computed from the training data.

Performance measures for classification include the true positive rate (TPR), true negative rate (TNR), weighted accuracy (wAcc) and the class error (clErr) as defined in the supplementary information.

The importance of each feature was estimated as the mean decrease in accuracy associated with dropping that variable from the model. This can be calculated for each class separately, the average of which forms the mean decrease accuracy overall. The Gini index is the mean decrease in Gini score associated with dropping the variable from the model, and thus the Gini score provides a measure of how well classes are separated.

Feature importance was investigated both for the full classification defined in Table 2, but also for separate sub‐classifications: Hemiptera (aphids) vs Coleoptera (beetles); within Hemiptera species; within Coleoptera species separately. For the latter two classifications, new training and validation datasets, satisfying the 70:30 split in each case, were defined.

Random forest models derived from different feature sets (Table S1) were compared with the out‐of‐bag error estimates and the predictive accuracy, calculated as the average proportion of correctly classified observations.

To simulate the process of identifying previously unidentified species, the random forest model was calibrated on all data excluding all observations of a single nominate species. This excluded species was then used as the validation dataset to form predictions. To investigate this process, the proportion of allocations to each species classification was extracted along with the maximal class probability. This process was repeated for each species in turn.

Random forests were fitted using the R package randomForest (Liaw & Wiener, 2002).

## Results

The average flight duration was 0.17 s across all taxa, translating to a speed of 0.41 m/s^−1^. Substantial variation in the flight duration was observed with interquartile range of 0.065–0.237 s and could be due to both the speed and direction of flight. Longer flights may involve spiralling as well as turning behaviour. Flight duration differed between species (F_5, 4773_ = 19.52, *p* < 0.001), with *M. persicae* (0.04 s; 1.75 m/s^−1^) and *P. chrysocephala* (0.076 s; 1.0 m/s^−1^) exhibiting shorter than average flight durations and thus higher speeds.

For each recording, up to 52 features were extracted (Table 1) and all show significant differences between species, on average (Table 3). Furthermore, with the exception of the amplitude index (of the raw audio), all features showed a greater variability among species than with either of the environmental covariates (largest F‐statistic is associated with species differences, Table 2). However, there is considerable variability within each feature reducing the chance that any one feature could in isolation discriminate between species without inclusion of additional features (Table S2).

**Table 3:** ANOVA results testing for differences between species for each individual feature

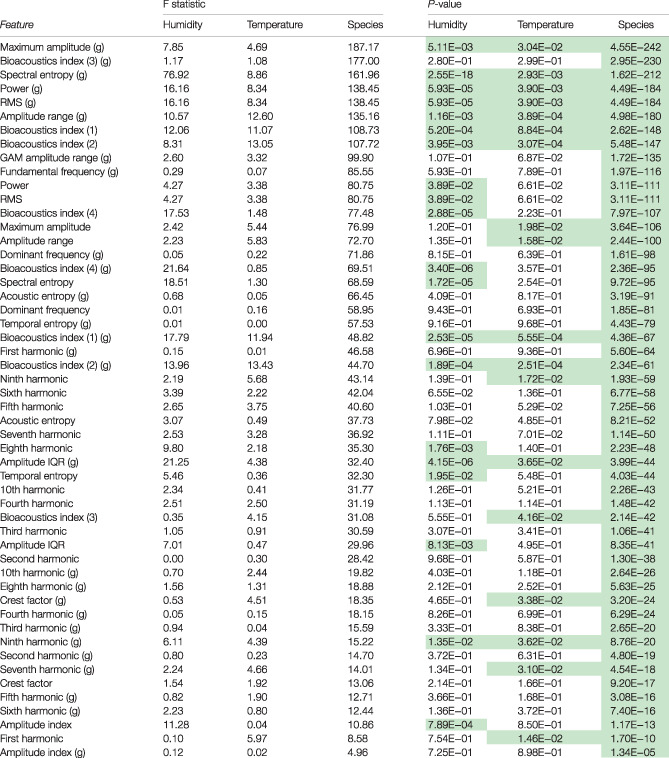

To adjust for confounding, the covariates humidity and temperature were included in the model. Significant terms are highlighted in green. F statistics reported are of type II. Features are ordered by the size of the F statistic associated with dropping species from the model (from largest to smallest). Where necessary, variables were transformed as listed in Table 1.

### Species classification

**Figure 2:**
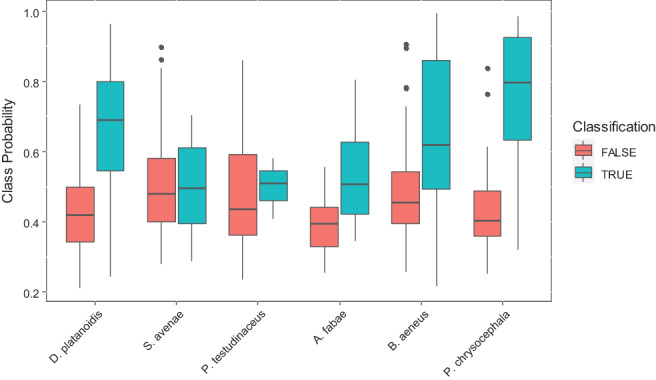
Species classification error rates. Box and whisker plots of the maximum class probability predicted for each observation in the validation dataset and split according to whether the classification was correct or not.

Classification to species level has varying levels of success with random forest models. An overall out‐of‐bag error rate of 20.62% (17.88%) on the training set and an error rate of 21.19% (17.75%) on the validation set including (excluding) observations with missing values suggest reasonable success in identifying individual species. A summary of the class‐specific error rates is given in the supplementary information (Figure S4). Random forest classification is better viewed in the ensemble framework within which it is derived. Figure 2 shows the distribution of the maximal class probability with an indication of whether the maximal probability coincided with the true underlying species. For those species with low misclassification rates (*D. platanoidis*, *B. aeneus*, *P. chrysocephala*), a direct correspondence is seen with high maximal class probability (a median of 0.69, 0.62, 0.80, respectively). Furthermore, for those observations of these species that are misclassified, the maximal class probability is lower (a median of 0.42, 0.46, 0.40), indicating greater uncertainty in the final classification. Although the certainty in the correct classification of *A. fabae* is lower (median of 0.51), there is still a pronounced increase in uncertainty when the classification is wrong (median of 0.39). By contrast, the certainty of classification for *S. avenae* and *P. testudinaceus* does not change depending upon whether the classification is correct or not (a median of 0.50, 0.51 for correct classifications and a median of 0.48, 0.43 for incorrect classifications).

### Features for classification

**Figure 3:**
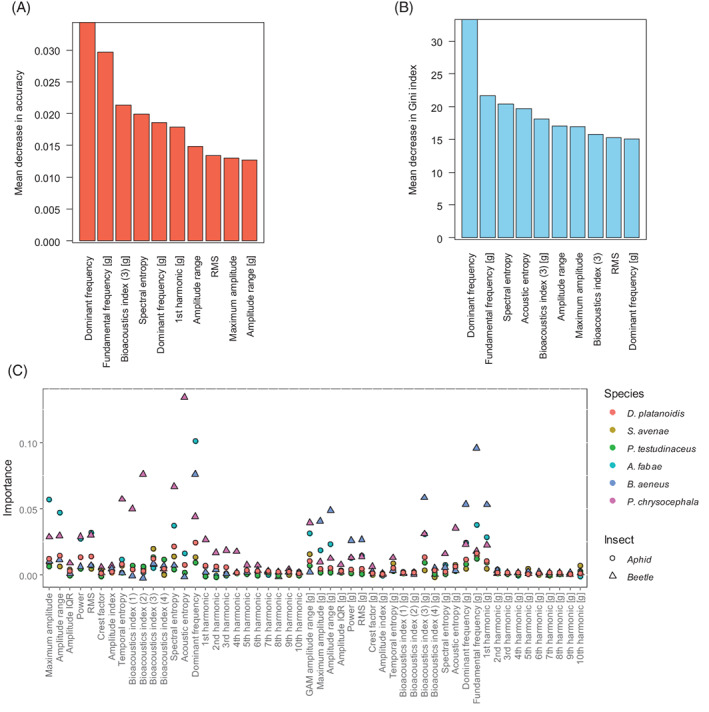
Species classification. (A,B) give the mean decrease in accuracy and Gini index, respectively, for the top 10 feature variables considered in the model (a complete set is shown in Fig. S5) and (C) presents the within species importance of each feature variable. Feature variables denoted by (g) are derived after a detrending step (see Fig. 1).

Figures 3 and S5 illustrate the relative importance of the different features in the classification model. The dominant frequency comes out top in both the accuracy (a measure of how well the prediction improves) and the Gini index (a measure of how well class separation improves) when the variable is included in the models. The fundamental frequency is a close second in terms of accuracy. It is clear that the higher‐order harmonics contribute little in terms of feature importance. By contrast, a number of summary indices of both the frequency and time domain are highlighted as important. These include the spectral and acoustic entropy, Bioacoustic Index (3) over 50–300 Hz, the RMS and power. Figure 3(C) shows that the importance of these features differs by species with the acoustic entropy important for identifying *P. chrysocephala* and the fundamental frequency important for *B. aeneus* and the dominant frequency for *A. fabae*.

### Harmonics alone are not enough

A comparison of out‐of‐bag error rates shows that classification improves when using all features of both the frequency and time domain compared with using specific subsets of feature variables (Fig. S6(A)). Specifically, the best out‐of‐bag accuracy rates on the validation set, where missing values are imputed, are seen when using all 52 features (78.9%) and when using the 27 features extracted after detrending (79.4%). A lower accuracy is seen when using the 25 features extracted before detrending (76.8%) and when using only the 20 harmonic features (76.8%). Marginal improvements are seen when supplementing the harmonic features with the additional frequency spectra indices (77.6%). Although the minimal feature sets of 3 and 5 chosen features result in lower accuracy (60.0% and 68.5%), the minimal set of chosen 14 features performs relatively well (75.6%). It can be seen that when imputation methods are used, the error rates tend to increase by about 2.5–4%.

Further investigation of the class‐specific error rates (Fig. S6(B)) shows that the high error rates of the minimal feature sets of 3 and 5 chosen features correspond with poor prediction of pollen beetles in particular. The predictive performance of black bean aphids increases in the feature sets restricted to the harmonics only, 75.0% class‐specific error rate compared with 44.2% in the full 52 feature set.

### Features for within‐order classification differ between order classification

**Figure 4:**
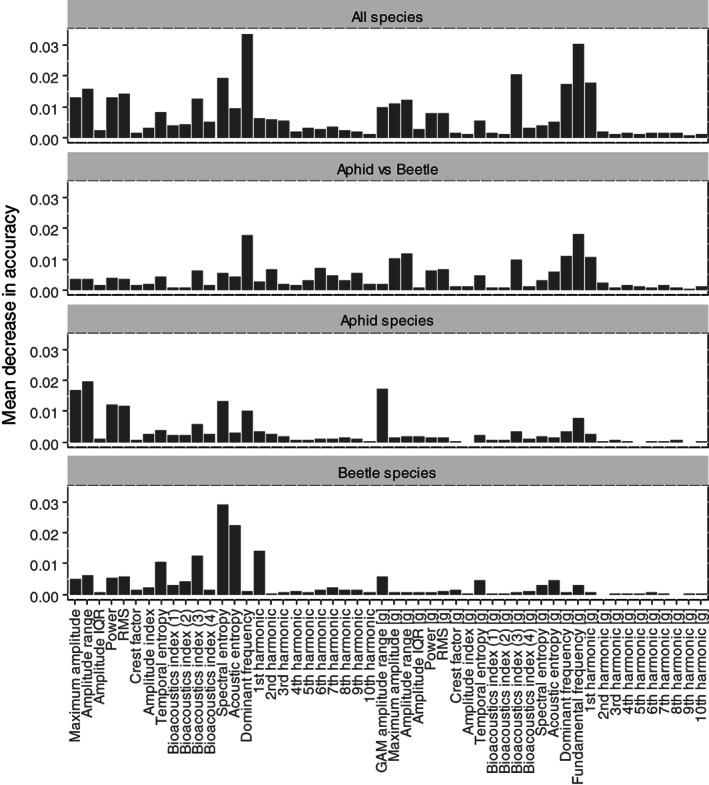
Comparison of class sets. The mean decrease in accuracy due to dropping each feature in turn from a model (using the complete feature set) classifying between all species, between aphids vs beetles, between aphid species and between beetle species.

Features important in classifying between Hemiptera and Coleoptera align very closely with those identified in the full model (Fig. 4). However, when data are restricted to a single order, differing patterns of feature importance are revealed. When focussed on aphid species only, the prominent features are the maximum amplitude, the amplitude range and the power or RMS (accuracy decrease), indicating a preference for features of the audio rather than the harmonics. The highly influential features of the full classification reduce to a mid or low level of importance in the within‐order classification. Similarly, focussing only on classifying between beetle species (albeit on a much smaller dataset), the key features of importance identified are the spectral and acoustic entropy and to a lesser extent, the bioacoustic index (at 50–300 Hz), the frequency of the most prominent harmonic and the temporal entropy, thus indicating a preference for features of the frequency spectra. Thus, harmonics such as the dominant frequency and fundamental frequency appear to be key in identifying between orders, but alternative features of the audio and spectrum are required to identify to a species level.

### Classifying unknown species results in less certain predictions

**Figure 5:**
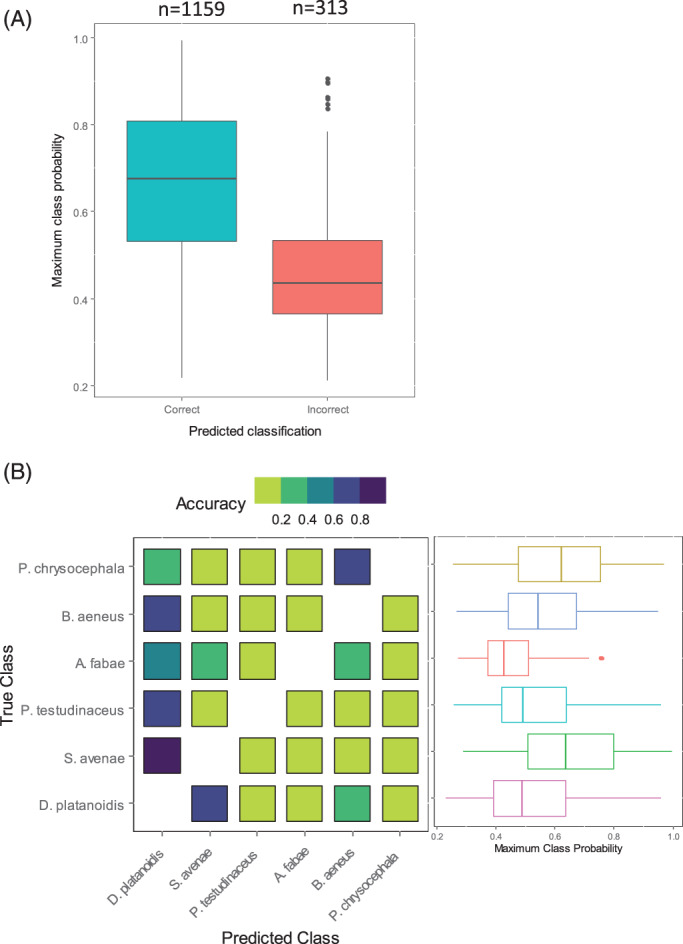
Identifying an unknown species. (A) Boxplots of the maximum class probability of the validation set for the full species model, separating between observations correctly and incorrectly classified. (B) Confusion matrix of each single species exclusion model showing the proportion of allocations to each of the known species. Boxplots of the maximum class probability are shown for each associated model.

In general, the class probability for a misclassified observation is lower than that for a correctly classified observation (Fig. 5(A)). Unobserved species are most commonly classified as sycamore aphids or pollen beetles, likely due to the larger number of observations in these two classes (Fig. 5(B)). Lower predictive certainty generally persists when investigating the model performance on predictions of a previously unobserved class (Fig. 5(B)). It is noticeable that when either cabbage stem flea beetles or English grain aphids are excluded from the training set, the class predictions remain relatively high, resulting in reasonable certainty that these species are in fact sycamore aphids or pollen beetles, respectively. When sycamore aphids are excluded from the training set, they are mostly allocated to the English grain aphid (62.3%) with some to the pollen beetle class (20.9%).

## Discussion

There is now a wealth of studies having developed classification models of insect flight (Moore, 1991; Chen *et al*., 2014; Potamitis, 2014; Ouyang *et al*., 2015; Potamitis *et al*., 2015; Kiskin *et al*., 2020), but relatively few have focussed on weak‐flying aphids and beetles (Moore & Miller, 2002). None that we know of have attempted to link morphological characteristics to acoustic properties with the exception of Rajabi *et al*. (2016)) who showed that the corrugated pattern of dragonfly wings explained differences between damsel and dragonfly wingbeat frequencies. The aim of this study has been to provide proof‐of‐concept for automatic detection methods of aphid and beetle pests *via* opto‐acoustic methods while also providing key insight into the drivers that will further this area of science. At first look, the error rates in misclassification in this study appear high at 18–20% and yet these insect pests are not only inaudible but their wingbeat rate is eight times smaller than the typical model species such as *Anopheles* mosquitoes (≈100 Hz v ≈ 800 Hz) and have a much weaker flight speed (0.41 m/s^−1^ vs ≈ 1 m/s^−1^) too (Potamitis *et al*., 2015). As sensors improve and more species are observed, it seems inevitable that the overall error rates will improve and yet we also anticipate specific species comparisons to remain a challenge. This is because of the close species similarity between aphid body plans, their small size (2–5 mm; body mass 1–13 mg) and their simple wings that do not affect the biomechanics of flight profoundly (*cf* dragonflies (Rajabi *et al*., 2016)). Successful classification to species level should not be the final endpoint, however. Rather the highly polyphagous nature of aphids and the differential risk such forms pose make it desirable to classify beyond species. Indeed, Hardie and Powell (2002)) show substantial variation in flight behaviour through video tracking technology between different forms of *A. fabae*. Although sensor technologies will undoubtedly improve, it is the view of the authors that black‐box classification of empirical data will always be limited in its scalability if not coupled with knowledge of morphology and if deployed in‐field, phenology.

At the highest taxonomic resolution, an ensemble of bioacoustics parameters and indices were used to distinguish between beetle and aphid species. Our models indicate that within a small selection of the Aphididae, measures of flight energy, particularly the maximum amplitude and the amplitude range, are more important than higher‐order harmonics even though stroke amplitude varies during flight (Tercel *et al*., 2018). Harmonics alone were shown to perform less well than conjectured by Moore and Miller (2002)) wherein they proved useful with neural networks. A lack of utility for harmonics in our study is perhaps surprising given that wingbeat frequency and the harmonics are functions of the physical size, shape, stiffness and mass of the wing as well as the wing muscles and stroke amplitude (Byrne *et al*., 1988; Tercel *et al*., 2018). However, because of their small size, aphids will likely incur greater relative drag and as a result of their small wings relative to body size will beat their wings comparatively faster than other insects and this appears to be an important discriminator (Byrne *et al*., 1988, Tercel *et al*., 2018). Such strengthening phenomena, discussed at length by Wootton (1981)) and shown to be the cause of variation in insect wing deformation between species, are also observed in birds, where higher wing loadings demand a more substantial humerus (Sullivan *et al*., 2019).

To further improve misclassification rates, a mechanistic understanding of wing acoustics is needed, and while a detailed investigation is beyond the scope of this study, some general observations are already profoundly clear; even with major differences in wing and flight apparatus, species are still misclassified between major groups, reducing overall model precision and accuracy; higher‐order wing beat harmonics do not play a major role in species discrimination, instead fundamental and dominant frequencies as well as audio are more high ranking.

Specifically concerning the first point, the forewings of beetles are hardened to form the elytra, such that the hindwing provides the energy and propulsion for flight and are not coupled to the elytra. Instead, both the elytra and hindwing beat in phase during flight, although the former have a smaller stroke angle (Brackenbury & Wang, 1995). Beetle wing venation is also modified to allow folding under the elytra when not in flight. Indeed, for both species of beetle studied here, individuals have poorly developed venation and therefore less stiffness (Kukalová‐Peck & Lawrence, 1993; Suzuki, 1994; Kukalová‐Peck & Lawrence, 2004). It can be clearly seen how flexible the chrysomelid beetle *Crepidodera aurata's* wing is without a rigid structure along the complete length of the hind wing (Nadein & Betz, 2016). Compare this flight apparatus with aphids that have coupled fore‐ and hind‐wings, no hardened wing casing and do not fold their wings on landing (Franielczyk‐Pietyra & Wegierek, 2017). Aphids, in contrast, have a thickened membrane on the forewing beyond the anterior costal margin, the pterostigma, that increases wing flap performance due to a stiffer leading edge that drives speed (Franielczyk‐Pietyra & Wegierek, 2017). Yet, aphid wings still retain a flexible wing membrane due to sparse venation, and this flexibility provides greater lift than a completely stiff wing (Mountcastle & Daniel, 2010). We therefore conjecture, ahead of any detailed study of wing bioacoustics, that differences in wing venation and morphology must play a minor role in generating unique wing harmonics for this group (or instead that if present, such differences cannot be detected in the current sensor through the exclusion of light principle), and, our models support this, stressing both the dominant and fundamental frequencies for splitting beetles and aphids, rather than higher harmonics or more complex indices relating to energy or mechanics. It is also possible one source of increased variability in higher‐order harmonics is the fact that wing movements may not be ‘clean’, for example, wings can touch each other or other parts of the body producing stridulations with ultrasonics or other harmonics, which would modulate the production of wing harmonics.

Our study shows that predicted mean (median) wingbeat frequencies for aphids at average temperature and humidity vary between species (Table S2; the exponentiated fundamental frequencies for six aphid species: *A. fabae* = 134 Hz (119 Hz); *D. platanoidis* = 104 Hz (95 Hz); *M. persicae* = 130 Hz (130 Hz); *P. testudinaceus* = 113 Hz (101 Hz); *R. padi* = 119 Hz (118 Hz); *S. avenae* = 106 Hz (99 Hz)) but fall within an expected range for hemipterans (90–152 Hz) (Tercel *et al*., 2018). The wingbeat frequencies of the cabbage stem flea beetle and pollen beetle are not known in the literature, but our values are not remarkably different from other confamilial species recorded by Tercel *et al*. (2018)) and Brackenbury and Wang (1995)) (Table S2; the exponentiated fundamental frequencies for *P. chrysocephala* = 121 Hz (119 Hz) *cf Oulema melanopus* 123 Hz Tercel *et al*. (2018) and *Chalcoides aurata* 118 Hz (Brackenbury & Wang, 1995). *B. aeneus* = 139 Hz (136 Hz)). These means are skewed somewhat when compared with the median, suggesting an increase in variation according to temperature. Previous studies have shown temperature to be positively correlated with the fundamental frequency, for example, female *Aedes aegypti* (L.) mosquitoes with a fundamental wingbeat frequency of ≈450–550 Hz, increase their flap rate ≈ 8–13 Hz per unit change in degree centigrade as the air becomes less dense, representing a rate of increase of 1.5–2.9% (Villarreal *et al*., 2017). ‘Frozen flight’ is another source of variation that can impact fundamental frequency estimates when the wingbeat is effectively zero (Thomas *et al*., 1977). Wing muscle autolysis in some of the aphids studied (*i.e. A. fabae*, *M. persicae and R. padi*), during which flight muscle breakdown removes the ability to fly, is yet another source of variation (Johnson, 1953; Leather *et al*., 1983). Wing muscle autolysis was particularly notable with *R. padi* that were largely grounded once in the flight arena. Despite these covariates, fundamental frequency remains key to discrimination, having a predictable relationship with wing area and to a lesser extent, body mass (Byrne *et al*., 1988; Tercel *et al*., 2018).

In emphasizing the data processing pipeline on feature extraction, we have been able to link indices of both the temporal and frequency domain to morphological characteristics. Thus, allowing us to gain understanding of the mechanisms contributing to differences in species flight behaviour. Such insight is unavailable in the convolutional neural network approach of inputting frequency spectra only. Furthermore, through our processing, we identified the importance of the flight movement (estimated *via* smoothing splines). Although this has previously been identified (Potamitis *et al*., 2015), to our knowledge it has not directly been incorporated into any classification algorithm. We have shown that relatively simple summaries of the temporal domain, such as the power indicative of the energy of a flight, contain important information for classification purposes, and perhaps explain why convolutional neural networks on wavelet transforms perform well (Kiskin *et al*., 2020) as wavelet transforms will account for both the time and frequency domain. This conceivably indicates that it is not only the short‐term flight behaviour such as wing‐flaps that are important for species identification but also the longer‐term trends in an insect flight. Future studies will investigate this further in the context of these weak‐flying agricultural pests.

Study design is one of the most important factors in any data collection activity. As with any study, there have been a number of limiting factors not least the high imbalance in observation numbers for each individual species. This does not appear to be uncommon in the literature as Moore and Miller (2002)) also had similarly imbalanced sample sizes ranging from 340 to 3325. Using balanced random forest approaches to account for the data imbalance, class‐specific error rates can be reduced albeit at the cost of higher out‐of‐bag error rates. Tuning these algorithms requires a trade‐off between false positive and false negative detections balanced across species. The optimal balance will depend on individual study aims; for instance, in‐field monitoring of pests would require a minimization of false negative detections of key agricultural pests, whereas for population monitoring it is preferable not to bias false detections to any one species. In this study, we have opted for the latter approach and to tune the algorithms aiming to balance class‐specific error rates. It remains the long‐term aim to deploy this technology in‐field enabling automatic insect pest‐detection at local spatial scales; however, further work is needed in the collation of robust labelled data. Furthermore, we envisage algorithmic development through the incorporation of prior knowledge, such as aphid migration patterns, as an essential component to obtain good accuracy in‐field.

## Authors' contributions

KLH developed the pre‐processing pipeline, analysed the data, devised the statistical approach and wrote the manuscript. AD collected the insect samples and ran the experiments. IP supplied the entomatic sensor and provided technical advice on the resulting data. JRB managed the project, secured the funding, and wrote the biological components of the manuscript. All authors read and approved the final manuscript.

## Supporting information


**Appendix** **S1:** Additional file 1: Supplementary Information
Supplementary information provides further details on the selection of features and the performance of the classification algorithms. Also included in this file are:
**Table S1.** detailing the chosen features for each random forest model
**Table S2.** containing the summary statistics of each extracted feature calculated per species.

**Appendix** **S2.** randomForest_dataSlice.R: script containing the random forest models for different data subsets (features and species).

**Appendix** **S3.** randomForest_speciesModel.R: script containing the random forest model for all species and associated analytics.

**Appendix** **S4.** Folder containing R scripts for running the random forest models and producing the presented analyses. Files included are: randomForest_tuning.R: script containing the tuning process of (a) the balanced random forest and (b) the tuning parameters of the random forest algorithm.

**Figure S1.** Photograph of the experimental setup where the opto‐acoustic sensor is contained within a large jar allowing aphids to fly freely through the sensor. Photographs of illustrative wings from *R. padi*, *D. platanoidis*, *B. aenus* and *P. chrysocephala*. Each tick mark on the scale bar is 0.1 mm.

**Figure S2.** Data processing. Figure illustrates how each recording is trimmed to remove periods of silence at the start or end of a recording. Lower panel is the audio recording, red lines are the threshold of ±0.0061 arbitrary amplitude units, above and below which sound is considered silence. The upper panel shows a box plot of the temporal indices exceeding this threshold. Audio is then trimmed to the whiskers of the boxplot defined as the largest (smallest) temporal index not exceeding three times the interquartile range away from the upper (lower) quartile, shown by the blue lines.

**Figure S3.** Identification of a minimal feature set. (A) The correlation matrix between features calculated after standardisation. (B) Dendrogram of a hierarchical cluster analysis using complete linkage on 1 – *r*, where *r* is the correlation matrix of the standardised feature set. Features are coloured according to cutting the dendrogram into (i) 3 groups, (ii) 5 groups (iii) 14 groups. Features deemed most representative of each group are indicated by the box.

**Figure S4.** Species classification error rates. (A) shows the class specific error rates where clErr is the class error rate (or 1 – true positive rate per class), TNR is the class specific true negative rate, TPR is the class specific true positive rate and wAcc is the weighted accuracy (wAcc = 0.5 × TNR + 0.5 × TPR). (B) shows the confusion matrices of classification predictions on the validation dataset, presented as a proportion per species.

**Figure S5.** Species classification. (A,B) give the mean decrease in accuracy and Gini index respectively for each feature variable considered in the model and (C) presents the within species importance of each feature variable. Feature variables denoted by (g) are derived after a detrending step (see Fig. 1)

**Figure S6.** Comparison of feature sets. (A) The mean accuracy rate on the out‐of‐bag predictions from the training set and on the validation set for both omitting and imputing observations with missing values. (B) the class specific error rates for each feature set for both omitting and imputing observations with missing values. Each model corresponds to a different subset of feature variables as detailed in Table 3.

## Data Availability

The datasets generated and/or analysed during the current study are publicly available with a DOI on the Rothamsted Research Repository (https://repository.rothamsted.ac.uk/) under a Creative Commons Attribution 4.0 Licence. Raw audio recordings are available at https://doi.org/10.23637/rothamsted.981w7 and extracted features at https://doi.org/10.23637/rothamsted.981w8. All codes used to analyse the data are available as supplementary materials.
