# Supplementary material for: Resolving the identification of weak‐flying insects during flight: a coupling between rigorous data processing and biology
Source: Agric For Entomol. 2021 Jun 2;23(4):489–505. doi: 10.1111/afe.12453 (PMC8596709; doi:10.1111/afe.12453)
Supplement: Supplementary file 1 — Appendix S1: Additional file 1: Supplementary Information Supplementary information provides further details on the selection of features and the performance of the classification algorithms. Also included in this file are: Table S1. detailing the chosen features for each random forest model Table S2. containing the summary statistics of each extracted feature calculated per species. [file AFE-23-489-s005.pdf]

Supplementary Information: **Resolving the identification of weak-flying insects during flight: a coupling between rigorous data processing and biology.**

Kirsty L. Hassall*, Alex Dye, Ilyas Potamitis & James R. Bell

*corresponding author: kirsty.hassall@rothamsted.ac.uk

Performance Measures for classification

Performance measures for classification include the true positive rate (TPR), true negative rate (TNR), weighted accuracy (wAcc) and the class error (clErr). Let TP_i be the number of true positives (the number of observations correctly identified for species *i*), TN_i be the number of true negatives (the number of observations correctly identified as not belonging to species *i*), FP_i be the number of false positives (the number of observations misidentified as species *i*) and FN_i be the number of false negatives (the number of observations misidentified as not belonging to species *i*), then,

$$TPR_i = TP_i/(TP_i + FN_i)$$

$$TNR_i = TN_i/(TPN_i + FP_i)$$

$$wAcc_i = 0.5 \times TNR_i + 0.5 \times TPR_i$$

$$clErr_i = 1 - TPR_i$$

The random forest algorithm identifies the probability that observation $X_j, j = 1, \ldots, n$ is allocated to species *i* as the number of trees which predict $X_j = i$ divided by the total number of trees grown. Thus, the maximal probability is defined by,

$$MP_j = max_i Prob(X_j = i)$$

with the predicted class defined by, $Pred_j = i$ such that $Prob(X_j = i) = MP_j$.

Selecting features

A total of 52 features were calculated for each audio recording. Seven different feature sets were considered and are shown in Table S1. The first set considers all 52 features. The second considers the 25 features calculated without detrending, whilst the third feature set considers the 27 features calculated on the detrended audio. Feature set 4 considers only the frequencies of the harmonic peaks, calculated both before and after signal detrending. Feature set 5 extends set 4 to also include the frequency indices such as the bioacoustics index, spectral entropy and dominant and fundamental frequencies. Feature sets 6, 7 and 8 consist of the representative features from a hierarchical cluster analysis with complete linkage on the correlation matrix of standardised features with 3, 5 and 14 clusters (Supplementary Figure S3). Representative features were defined to be the feature closest to the cluster centroid

Species classification in unbalanced sample sizes

Classification to species level have varying levels of success with random forest models. An overall out-of-bag error rate of 20.62% (17.88%) on the training set and an error rate of 21.19% (17.75%) on the validation set including (excluding) observations with missing values suggest reasonable success in identifying individual species. However, due to the high imbalance in sample sizes of each species, it is more informative to investigate the class specific error rates. It is clear from Figure S4A that the classification of *P. testudinaceus*, is very poor with a misclassification rate of 87%, however, it should be recalled that the Maple aphid only accounts for 2% of the observations. Similarly, *S. avenae* accounting for 6% of the observations also demonstrates poor misclassification at 65%. However, *A. fabae* accounting for only 3% of the data misclassifies at a rate of 44% demonstrating the misclassification is not solely due to the unbalanced sample sizes. Furthermore, Figure S4B shows the confusion matrix in predictions on the validation set where it can be seen that *S. avenae* and *P. testudinaceus* are more likely to be classified as *D. platanoidis* than they are to be correctly identified. However, classification is better viewed in the probabilistic framework within which it's derived. Figure

2 shows the distribution of the maximal class probability with an indication of whether the maximal probability coincided with the true underlying species. For those species with low misclassification rates (*D. platanoidis, B. aeneus, P. chrysocephala*), a direct correspondence is seen with high maximal class probability (a median of 0.69, 0.62, 0.80, respectively). Furthermore, for those observations of these species that are misclassified, the maximal class probability is lower (a median of 0.42, 0.46, 0.40) indicating greater uncertainty in the final classification. Although the certainty in the correct classification of *A. fabae* is lower (median of 0.51), there is still a pronounced increase in uncertainty when the classification is wrong (median of 0.39). In contrast, the certainty of classification for *S. avenae* and *P. testudinaceus* does not change depending upon whether the classification is correct or not (a median of 0.50, 0.51 for correct classifications and a median of 0.48, 0.43 for incorrect classifications).

Table S1 Feature sets. The list of features included in each random forest model

	Maximum amplitude	Amplitude range	Amplitude IQR	Power	RMS	Crest factor	Amplitude index	Temporal entropy	Bioacoustics index (1)	Bioacoustics index (2)	Bioacoustics index (3)	Bioacoustics index (4)	Spectral entropy	Acoustic entropy	Dominant frequency	1st harmonic	2nd harmonic	3rd harmonic	4th harmonic	5th harmonic	6th harmonic	7th harmonic	8th harmonic	9th harmonic	10th harmonic
	audio	audio	audio	audio	audio	audio	audio	audio	frequency	frequency	frequency	frequency	frequency	audio-frequency	frequency	harmonics	harmonics	harmonics	harmonics	harmonics	harmonics	harmonics	harmonics	harmonics	harmonics
**1**	■	■	■	■	■	■	■	■	■	■	■	■	■	■	■	■	■	■	■	■	■	■	■	■	■
**2**	■	■	■	■	■	■	■	■	■	■	■	■	■	■	■	■	■	■	■	■	■	■	■	■	■
**3**																									
**4**																■	■	■	■	■	■	■	■	■	■
**5**									■	■	■	■	■		■	■	■	■	■	■	■	■	■	■	■
**6**				■																					
**7**				■							■		■												
**8**		■	■			■					■		■		■	■				■					

	GAM amplitude range	Maximum amplitude [g]	Amplitude range [g]	Amplitude IQR [g]	Power [g]	RMS [g]	Crest factor [g]	Amplitude index [g]	Temporal entropy [g]	Bioacoustics index (1) [g]	Bioacoustics index (2) [g]	Bioacoustics index (3) [g]	Bioacoustics index (4) [g]	Spectral entropy [g]	Acoustic entropy [g]	Dominant frequency [g]	1st harmonic [g]	2nd harmonic [g]	3rd harmonic [g]	4th harmonic [g]	5th harmonic [g]	6th harmonic [g]	7th harmonic [g]	8th harmonic [g]	9th harmonic [g]	10th harmonic [g]	Fundamental frequency [g]
	audio	audio	audio	audio	audio	audio	audio	audio	audio	frequency	frequency	frequency	frequency	frequency	audio-frequency	frequency	harmonics	harmonics	harmonics	harmonics	harmonics	harmonics	harmonics	harmonics	harmonics	harmonics	harmonics
**1**	■	■	■	■	■	■	■	■	■	■	■	■	■	■	■	■	■	■	■	■	■	■	■	■	■	■	■
**2**																											
**3**	■	■	■	■	■	■	■	■	■	■	■	■	■	■	■	■	■	■	■	■	■	■	■	■	■	■	■
**4**																	■	■	■	■	■	■	■	■	■	■	
**5**										■	■	■	■	■		■	■	■	■	■	■	■	■	■	■	■	■
**6**															■							■					
**7**									■													■					
**8**									■	■						■		■		■				■			

Table S2 Summary statistics of each feature calculated per species. SE is the residual standard error of the linear models fitted in Table 4 and the predicted means are shown on the scale of analysis where applicable transformations are listed in Table 1.

			Mean (predicted at the average temperature and humidity)							
Feature	**N**	**SE**	*A. fabae*	*B. aeneus*	*D. platanoidis*	*M. persicae*	*P. testudinaceus*	*P. chrysocephala*	*R. padi*	*S. avenae*
**Maximum amplitude**	4928	0.69858	-8.529	-7.529	-7.721	-8.55	-8.159	-7.109	-8.696	-8.094
**Amplitude range**	4928	0.73322	-8.002	-7	-7.2	-7.994	-7.719	-6.564	-8.126	-7.551
**Amplitude IQR**	4928	0.7852	-9.35	-8.998	-9.125	-9.157	-9.419	-8.443	-9.53	-9.362
**Crest factor**	4928	0.26612	0.9522	1.073	1.065	0.8058	1.065	0.9235	0.9581	1.072
**Power**	4928	1.3713	-18.96	-17.2	-17.57	-18.71	-18.45	-16.07	-19.31	-18.33
**RMS**	4928	0.68563	-9.481	-8.602	-8.785	-9.356	-9.224	-8.033	-9.654	-9.166
**Dominant frequency**	4884	0.079167	0.3598	0.3606	0.2995	0.3014	0.3365	0.3701	0.3431	0.3138
**Bioacoustics index (1)**	4928	0.62789	2.881	3.391	3.07	3.125	2.89	4.16	2.515	2.89
**Bioacoustics index (2)**	4928	0.60057	2.741	3.199	2.957	2.896	2.762	4.045	2.428	2.789
**Bioacoustics index (3)**	4928	0.22327	0.6142	0.6427	0.7757	0.928	0.7813	0.777	0.6522	0.6646
**Bioacoustics index (4)**	4928	1.4334	5.019	6.032	4.849	6.245	4.799	6.31	5.119	4.88
**Amplitude index**	4928	1.0861	-19.32	-19.03	-19.13	-21.06	-19.06	-19.47	-19.37	-19.25
**Spectral entropy**	4928	0.063997	0.8327	0.7667	0.7705	0.7968	0.7967	0.6958	0.8564	0.8104
**Temporal entropy**	4928	0.12637	0.29	0.2389	0.2612	0.123	0.2753	0.1369	0.3032	0.2815
**Acoustic entropy**	4928	0.10631	0.2459	0.1854	0.2037	0.09784	0.2233	0.09705	0.2614	0.2323
**1st harmonic**	4928	0.029872	0.02977	0.01591	0.01509	0.02396	0.02077	0.0256	0.01375	0.01247
**2nd harmonic**	4928	0.091842	0.08728	0.09301	0.05804	0.09655	0.07806	0.1381	0.02532	0.0641
**3rd harmonic**	4928	0.16137	0.1386	0.1525	0.1017	0.2474	0.1519	0.2581	0.07728	0.1244
**4th harmonic**	4928	0.25456	0.1767	0.2412	0.1548	0.3536	0.2329	0.3983	0.0531	0.2048
**5th harmonic**	4928	0.35765	0.2631	0.3468	0.2133	0.5162	0.354	0.6102	0.2021	0.27
**6th harmonic**	4928	0.44321	0.3285	0.4715	0.2936	0.6364	0.415	0.7867	0.07886	0.3574
**7th harmonic**	4928	0.5157	0.4478	0.5451	0.3817	0.6457	0.4356	0.9567	0.2988	0.4243
**8th harmonic**	4928	0.60969	0.5121	0.6927	0.4969	1.245	0.5787	1.113	0.3268	0.496
**9th harmonic**	4928	0.65528	0.6707	0.8546	0.6047	1.263	0.6427	1.32	0.6956	0.5186

**10th harmonic**	4928	0.7291	0.8153	0.9839	0.7677	1.081	0.7873	1.468	0.717	0.6375
**GAM amplitude range [g]**	4317	0.70492	-3.305	-2.208	-2.391	-3.353	-2.787	-1.339	-3.662	-2.877
**Maximum amplitude [g]**	4317	0.000103	0.0001015	0.0002335	0.0001358	0.0001068	0.0001591	0.000378	8.09E-05	0.0001187
**Amplitude range [g]**	4317	0.51611	-8.737	-7.919	-8.438	-8.789	-8.37	-7.665	-8.952	-8.532
**Amplitude IQR [g]**	4317	0.56116	-10.62	-10.66	-10.65	-10.55	-10.51	-9.953	-11.06	-10.59
**Crest factor [g]**	4317	0.24133	1.269	1.44	1.347	1.239	1.329	1.242	1.357	1.355
**Power [g]**	4317	0.94418	-21.17	-19.9	-20.74	-21.21	-20.54	-19	-21.76	-20.94
**RMS [g]**	4317	0.47209	-10.59	-9.952	-10.37	-10.6	-10.27	-9.5	-10.88	-10.47
**Fundamental frequency [g]**	4143	0.24834	-2.011	-1.972	-2.261	-2.039	-2.172	-2.114	-2.131	-2.24
**Dominant frequency [g]**	4317	0.051175	0.3627	0.3723	0.3182	0.3517	0.3446	0.3433	0.3778	0.3267
**Bioacoustics index (1) [g]**	4317	0.68034	2.341	2.785	2.368	2.365	2.961	2.964	1.893	2.235
**Bioacoustics index (2) [g]**	4317	0.65212	2.224	2.616	2.256	2.296	2.784	2.851	1.785	2.136
**Bioacoustics index (3) [g]**	4317	0.22899	0.6863	0.8016	0.4874	0.9177	0.6027	0.8931	0.6747	0.5364
**Bioacoustics index (4) [g]**	4317	1.3109	4.883	5.943	4.785	5.094	5.271	5.998	4.953	4.742
**Amplitude index [g]**	4317	1.4618	-23.22	-23.56	-23.39	-23.85	-23.45	-23.89	-23.46	-23.15
**Spectral entropy [g]**	4317	0.030443	0.9204	0.8867	0.911	0.9446	0.8943	0.843	0.9416	0.9203
**Temporal entropy [g]**	4317	0.11677	0.2736	0.1861	0.2521	0.09699	0.2464	0.1189	0.2444	0.2903
**Acoustic entropy [g]**	4317	0.1058	0.2509	0.1644	0.2298	0.09183	0.22	0.1019	0.2295	0.2671
**1st harmonic [g]**	4317	0.045413	0.1352	0.1423	0.1034	0.1246	0.1245	0.1198	0.145	0.1093
**2nd harmonic [g]**	4317	0.12347	0.2234	0.1877	0.1445	0.2639	0.1655	0.2046	0.1876	0.1519
**3rd harmonic [g]**	4317	0.20092	0.293	0.2647	0.1903	0.2736	0.2839	0.2946	0.2613	0.194
**4th harmonic [g]**	4317	0.2968	0.3334	0.3443	0.2532	0.253	0.3171	0.4667	0.6407	0.2346
**5th harmonic [g]**	4317	0.3797	0.3803	0.4507	0.3225	0.5484	0.2915	0.5065	0.3962	0.3113
**6th harmonic [g]**	4317	0.46519	0.4954	0.5542	0.4158	0.2192	0.3984	0.6312	1.142	0.3703
**7th harmonic [g]**	4317	0.54075	0.5621	0.6871	0.5051	0.7391	0.4231	0.7742	1.083	0.4816
**8th harmonic [g]**	4317	0.60709	0.6759	0.8571	0.6136	0.5627	0.6176	0.9453	0.8483	0.4981
**9th harmonic [g]**	4317	0.67081	0.7499	0.9476	0.7469	0.999	0.6054	1.113	1.101	0.6197
**10th harmonic [g]**	4317	0.7227	0.9635	1.124	0.8787	1.846	0.9592	1.311	1.435	0.6595

	Median (Lower quartile, Upper quartile)			
**Feature**	*A. fabae*	*B. aeneus*	*D. platanoidis*	*M. persicae*
**Maximum amplitude**	-8.637 (-8.935,-8.261)	-7.424 (-7.998,-6.995)	-7.645 (-8.178,-7.194)	-8.398 (-8.923,-8.263)
**Amplitude range**	-8.076 (-8.441,-7.683)	-6.91 (-7.476,-6.416)	-7.109 (-7.672,-6.64)	-7.95 (-8.29,-7.61)
**Amplitude IQR**	-9.516 (-9.813,-9.051)	-9.18 (-9.604,-8.543)	-9.181 (-9.638,-8.67)	-9.275 (-9.626,-8.658)
**Crest factor**	0.9503 (0.7783,1.109)	1.064 (0.8277,1.29)	1.036 (0.8611,1.232)	0.7352 (0.6541,0.809)
**Power**	-19.22 (-19.86,-18.44)	-17.19 (-18.14,-16.18)	-17.5 (-18.45,-16.55)	-18.72 (-19.67,-17.83)
**RMS**	-9.611 (-9.931,-9.221)	-8.593 (-9.072,-8.09)	-8.75 (-9.225,-8.277)	-9.359 (-9.834,-8.914)
**Dominant frequency**	0.3452 (0.3187,0.3634)	0.3624 (0.3217,0.384)	0.2898 (0.2693,0.3094)	0.2778 (0.2605,0.3342)
**Bioacoustics index (1)**	2.737 (2.398,3.095)	3.308 (2.913,3.726)	3.074 (2.715,3.445)	2.991 (2.635,3.658)
**Bioacoustics index (2)**	2.622 (2.318,2.918)	3.104 (2.709,3.538)	2.95 (2.622,3.317)	2.909 (2.502,3.117)
**Bioacoustics index (3)**	0.5997 (0.4994,0.7798)	0.6377 (0.5186,0.7625)	0.7662 (0.634,0.9085)	0.917 (0.8531,1.075)
**Bioacoustics index (4)**	4.766 (3.875,5.639)	5.672 (4.821,6.8)	4.674 (4.002,5.585)	5.882 (4.913,7.613)
**Amplitude index**	-19.4 (-20.05,-18.95)	-19.05 (-19.62,-18.53)	-19.13 (-19.64,-18.67)	-20.91 (-21.47,-20.7)
**Spectral entropy**	0.8542 (0.8084,0.8808)	0.7683 (0.7272,0.8119)	0.766 (0.7165,0.8156)	0.7816 (0.7585,0.8325)
**Temporal entropy**	0.2529 (0.1718,0.366)	0.2082 (0.1432,0.295)	0.2244 (0.1623,0.3347)	0.1223 (0.1199,0.1312)
**Acoustic entropy**	0.2128 (0.1336,0.3204)	0.1618 (0.1055,0.236)	0.1727 (0.1193,0.267)	0.09745 (0.09383,0.1023)
**1st harmonic**	0.01074 (0.007812,0.0166)	0.0127 (0.006836,0.01953)	0.0127 (0.007812,0.01855)	0.02344 (0.02148,0.02441)
**2nd harmonic**	0.04199 (0.01562,0.1172)	0.07129 (0.02344,0.1357)	0.04883 (0.01953,0.08301)	0.07715 (0.06787,0.1216)
**3rd harmonic**	0.1133 (0.04004,0.1484)	0.1318 (0.04785,0.1831)	0.08691 (0.04102,0.1279)	0.127 (0.1113,0.1606)
**4th harmonic**	0.124 (0.07324,0.2314)	0.1587 (0.09131,0.2795)	0.1162 (0.07031,0.1826)	0.1875 (0.1558,0.2407)
**5th harmonic**	0.1562 (0.09277,0.2715)	0.2354 (0.114,0.406)	0.1592 (0.08105,0.2432)	0.2334 (0.2114,0.312)
**6th harmonic**	0.2148 (0.1035,0.3584)	0.3003 (0.1384,0.5781)	0.1846 (0.09473,0.3057)	0.2979 (0.2378,0.4614)
**7th harmonic**	0.2451 (0.1172,0.5137)	0.3745 (0.1538,0.7034)	0.2373 (0.1143,0.374)	0.373 (0.2847,0.9868)
**8th harmonic**	0.3262 (0.1328,0.5957)	0.499 (0.196,1.089)	0.2725 (0.1445,0.4995)	1.209 (0.5522,1.806)
**9th harmonic**	0.3447 (0.1777,0.9619)	0.6709 (0.2769,1.417)	0.3359 (0.1729,0.7437)	1.333 (0.6973,1.708)
**10th harmonic**	0.4824 (0.2217,1.402)	0.8579 (0.3281,1.593)	0.4424 (0.2188,1.186)	1.111 (0.6562,1.523)

**GAM amplitude range [g]**	-3.396 (-3.8,-2.951)	-2.166 (-2.656,-1.66)	-2.308 (-2.826,-1.845)	-3.671 (-3.771,-3.109)
**Maximum amplitude [g]**	8.007e-05 (6.14e-05,0.0001062)	0.0002019 (0.0001316,0.0003018)	0.0001221 (8.552e-05,0.0001709)	6.455e-05 (5.467e-05,0.0001366)
**Amplitude range [g]**	-8.864 (-9.117,-8.598)	-7.904 (-8.334,-7.513)	-8.424 (-8.777,-8.085)	-9.097 (-9.218,-8.534)
**Amplitude IQR [g]**	-10.76 (-10.96,-10.52)	-10.8 (-11.2,-10.33)	-10.65 (-10.97,-10.33)	-10.57 (-10.62,-10.49)
**Crest factor [g]**	1.266 (1.101,1.411)	1.435 (1.252,1.62)	1.333 (1.178,1.5)	1.057 (0.943,1.43)
**Power [g]**	-21.48 (-21.75,-21.04)	-19.94 (-20.73,-19.29)	-20.76 (-21.33,-20.15)	-21.41 (-21.55,-20.98)
**RMS [g]**	-10.74 (-10.87,-10.52)	-9.969 (-10.36,-9.645)	-10.38 (-10.66,-10.07)	-10.71 (-10.77,-10.49)
**Fundamental frequency [g]**	-2.125 (-2.169,-1.998)	-1.998 (-2.064,-1.928)	-2.351 (-2.431,-2.155)	-2.039 (-2.104,-1.974)
**Dominant frequency [g]**	0.348 (0.3348,0.3648)	0.3671 (0.3452,0.3865)	0.3014 (0.2898,0.3233)	0.3409 (0.3351,0.3637)
**Bioacoustics index (1) [g]**	2.108 (1.88,2.452)	2.577 (2.228,3.007)	2.342 (2.009,2.736)	2.534 (2.129,2.67)
**Bioacoustics index (2) [g]**	2.014 (1.819,2.286)	2.399 (2.083,2.841)	2.234 (1.907,2.603)	2.483 (2.069,2.594)
**Bioacoustics index (3) [g]**	0.6859 (0.5986,0.8167)	0.8112 (0.6103,0.97)	0.4322 (0.3405,0.5661)	0.9567 (0.8994,0.9577)
**Bioacoustics index (4) [g]**	4.536 (3.829,5.33)	5.624 (4.79,6.592)	4.644 (3.998,5.5)	5.236 (4.892,5.478)
**Amplitude index [g]**	-23.37 (-23.98,-22.86)	-23.5 (-24.23,-22.93)	-23.49 (-24.25,-22.83)	-23.33 (-24.14,-23.31)
**Spectral entropy [g]**	0.9356 (0.9211,0.943)	0.8918 (0.8717,0.9168)	0.9121 (0.8905,0.9299)	0.9411 (0.9371,0.9487)
**Temporal entropy [g]**	0.2352 (0.1434,0.3594)	0.1679 (0.1139,0.2396)	0.2231 (0.1505,0.3272)	0.09958 (0.09426,0.101)
**Acoustic entropy [g]**	0.2204 (0.1339,0.3377)	0.1486 (0.1005,0.2171)	0.2033 (0.1362,0.2973)	0.09372 (0.08938,0.0946)
**1st harmonic [g]**	0.1211 (0.1121,0.1331)	0.1348 (0.1191,0.1494)	0.09082 (0.08398,0.1045)	0.1162 (0.1123,0.1328)
**2nd harmonic [g]**	0.1445 (0.1113,0.2278)	0.1455 (0.1201,0.2388)	0.1338 (0.09473,0.1729)	0.2979 (0.2285,0.3164)
**3rd harmonic [g]**	0.2148 (0.1184,0.3064)	0.2227 (0.1313,0.2842)	0.1685 (0.1182,0.2246)	0.1953 (0.1924,0.3174)
**4th harmonic [g]**	0.2344 (0.1335,0.3574)	0.2646 (0.1523,0.4136)	0.1943 (0.1436,0.2695)	0.2432 (0.2354,0.2622)
**5th harmonic [g]**	0.2671 (0.1655,0.3999)	0.3389 (0.1855,0.5435)	0.2393 (0.1631,0.334)	0.2705 (0.2568,0.6841)
**6th harmonic [g]**	0.3262 (0.2031,0.5959)	0.4121 (0.2188,0.7124)	0.2681 (0.1719,0.4053)	0.1777 (0.1484,0.25)
**7th harmonic [g]**	0.3325 (0.1931,0.6475)	0.498 (0.2642,0.9751)	0.312 (0.1963,0.5042)	0.667 (0.501,0.9028)
**8th harmonic [g]**	0.4321 (0.2351,0.8965)	0.667 (0.3037,1.37)	0.3691 (0.2314,0.7053)	0.5029 (0.4692,0.5996)
**9th harmonic [g]**	0.4409 (0.1951,1.198)	0.8223 (0.373,1.489)	0.4443 (0.2585,1.068)	0.374 (0.3174,1.312)
**10th harmonic [g]**	0.5801 (0.3186,1.61)	1.03 (0.4448,1.711)	0.5786 (0.3154,1.388)	2.278 (1.385,2.488)

Feature	Median (Lower quartile, Upper quartile)			
	P. testudinaceus	*P. chrysocephala*	*R. padi*	*S. avenae*
**Maximum amplitude**	-8.14 (-8.597,-7.765)	-6.73 (-7.687,-6.257)	-8.687 (-8.756,-8.586)	-8.098 (-8.542,-7.706)
**Amplitude range**	-7.765 (-8.21,-7.24)	-6.098 (-7.065,-5.682)	-8.093 (-8.22,-7.976)	-7.529 (-8.012,-7.135)
**Amplitude IQR**	-9.459 (-9.942,-9.014)	-8.176 (-9.267,-7.481)	-9.943 (-10.01,-9.132)	-9.431 (-9.84,-8.883)
**Crest factor**	1.078 (0.8749,1.239)	0.8531 (0.7592,1.033)	1.003 (0.7371,1.238)	1.06 (0.8598,1.234)
**Power**	-18.46 (-19.51,-17.73)	-15.31 (-17.15,-14.44)	-19.35 (-20.01,-18.5)	-18.43 (-19.27,-17.57)
**RMS**	-9.231 (-9.756,-8.867)	-7.655 (-8.573,-7.218)	-9.676 (-10,-9.251)	-9.213 (-9.633,-8.785)
**Dominant frequency**	0.3014 (0.2577,0.3445)	0.3584 (0.3026,0.3956)	0.3409 (0.3212,0.3617)	0.3062 (0.2847,0.3229)
**Bioacoustics index (1)**	2.852 (2.438,3.328)	4.367 (3.437,4.936)	2.423 (2.075,2.775)	2.814 (2.466,3.182)
**Bioacoustics index (2)**	2.768 (2.284,3.181)	4.233 (3.341,4.788)	2.384 (2.063,2.689)	2.7 (2.417,2.995)
**Bioacoustics index (3)**	0.7561 (0.5443,1.009)	0.7092 (0.5318,1.014)	0.6186 (0.5974,0.7159)	0.6217 (0.5009,0.8162)
**Bioacoustics index (4)**	4.585 (3.716,5.523)	6.593 (5.067,7.586)	4.509 (3.849,5.638)	4.819 (4.145,5.664)
**Amplitude index**	-18.97 (-19.48,-18.59)	-19.21 (-19.94,-18.79)	-19.4 (-19.88,-19.17)	-19.19 (-19.79,-18.77)
**Spectral entropy**	0.8066 (0.7538,0.8407)	0.6702 (0.6415,0.7136)	0.8739 (0.8349,0.9022)	0.8179 (0.769,0.8555)
**Temporal entropy**	0.2288 (0.1741,0.3251)	0.1071 (0.0969,0.1547)	0.2883 (0.2588,0.3181)	0.2656 (0.1661,0.3809)
**Acoustic entropy**	0.1754 (0.1338,0.2663)	0.07308 (0.06627,0.1032)	0.2486 (0.2238,0.2889)	0.2182 (0.1263,0.3151)
**1st harmonic**	0.01172 (0.007812,0.01758)	0.02637 (0.01953,0.02832)	0.008789 (0.007568,0.009521)	0.01123 (0.007812,0.01855)
**2nd harmonic**	0.03711 (0.01855,0.09082)	0.1284 (0.08154,0.156)	0.021 (0.01831,0.02417)	0.03076 (0.01392,0.08691)
**3rd harmonic**	0.07812 (0.04395,0.1562)	0.2178 (0.1301,0.3037)	0.1162 (0.02808,0.1309)	0.08496 (0.02539,0.1267)
**4th harmonic**	0.1123 (0.06055,0.2061)	0.3174 (0.1936,0.4031)	0.06836 (0.03418,0.1331)	0.1064 (0.06519,0.1902)
**5th harmonic**	0.1377 (0.08496,0.292)	0.4189 (0.2751,0.6208)	0.1602 (0.04639,0.2395)	0.126 (0.08032,0.2146)
**6th harmonic**	0.1758 (0.0957,0.3438)	0.5308 (0.3298,1.156)	0.09082 (0.04614,0.1399)	0.1841 (0.09473,0.2959)
**7th harmonic**	0.2188 (0.1152,0.3994)	0.7129 (0.427,1.429)	0.3291 (0.04663,0.4175)	0.1982 (0.09985,0.3701)
**8th harmonic**	0.252 (0.1338,0.8789)	0.9043 (0.4648,1.605)	0.1807 (0.1013,0.4641)	0.2188 (0.1187,0.4368)
**9th harmonic**	0.2998 (0.1533,1.074)	1.266 (0.6477,1.922)	0.3882 (0.09717,1.545)	0.293 (0.1414,0.488)
**10th harmonic**	0.459 (0.1846,1.37)	1.375 (0.8262,1.992)	0.4722 (0.1599,1.407)	0.3237 (0.1799,0.7134)

**GAM amplitude range [g]**	-2.744 (-3.27,-2.278)	-1.12 (-1.737,-0.7614)	-3.764 (-4.013,-3.569)	-2.873 (-3.329,-2.425)
**Maximum amplitude [g]**	0.0001341 (9.616e-05,0.0001659)	0.0003021 (0.0001379,0.0004716)	6.981e-05 (5.913e-05,9.082e-05)	0.0001079 (7.802e-05,0.0001428)
**Amplitude range [g]**	-8.397 (-8.69,-8.116)	-7.474 (-8.271,-7.1)	-9.047 (-9.145,-8.758)	-8.546 (-8.871,-8.28)
**Amplitude IQR [g]**	-10.56 (-10.83,-10.28)	-9.808 (-10.71,-9.122)	-11.14 (-11.19,-11.01)	-10.55 (-10.82,-10.39)
**Crest factor [g]**	1.318 (1.154,1.5)	1.201 (1.115,1.318)	1.381 (1.218,1.399)	1.354 (1.184,1.477)
**Power [g]**	-20.68 (-21.07,-20.17)	-18.69 (-20.46,-17.56)	-21.91 (-21.94,-21.69)	-20.98 (-21.44,-20.55)
**RMS [g]**	-10.34 (-10.54,-10.08)	-9.346 (-10.23,-8.781)	-10.95 (-10.97,-10.84)	-10.49 (-10.72,-10.27)
**Fundamental frequency [g]**	-2.29 (-2.384,-1.985)	-2.125 (-2.303,-1.964)	-2.14 (-2.14,-2.11)	-2.315 (-2.375,-2.225)
**Dominant frequency [g]**	0.3187 (0.2948,0.375)	0.3395 (0.3125,0.3698)	0.3604 (0.338,0.3631)	0.3141 (0.2997,0.3311)
**Bioacoustics index (1) [g]**	2.567 (2.244,2.919)	2.898 (2.333,3.322)	1.783 (1.771,1.844)	2.134 (1.871,2.448)
**Bioacoustics index (2) [g]**	2.441 (2.119,2.831)	2.759 (2.226,3.244)	1.755 (1.72,1.8)	2.014 (1.782,2.342)
**Bioacoustics index (3) [g]**	0.5356 (0.4243,0.7379)	0.9862 (0.5682,1.154)	0.7043 (0.6045,0.7557)	0.5086 (0.4038,0.6197)
**Bioacoustics index (4) [g]**	4.803 (4.196,5.866)	5.911 (4.986,6.974)	4.436 (3.93,4.596)	4.749 (4.049,5.524)
**Amplitude index [g]**	-23.57 (-24.38,-22.92)	-23.81 (-24.52,-23.22)	-22.85 (-24.08,-22.31)	-23.38 (-24.11,-22.62)
**Spectral entropy [g]**	0.9033 (0.8804,0.9229)	0.8421 (0.7955,0.8809)	0.9523 (0.9479,0.9548)	0.9203 (0.9067,0.9338)
**Temporal entropy [g]**	0.2159 (0.1552,0.3038)	0.1016 (0.08044,0.1269)	0.2522 (0.227,0.2844)	0.2803 (0.1868,0.3668)
**Acoustic entropy [g]**	0.1966 (0.1391,0.2785)	0.08368 (0.06969,0.1038)	0.2408 (0.2137,0.2696)	0.2568 (0.1714,0.3358)
**1st harmonic [g]**	0.1016 (0.08691,0.1406)	0.1152 (0.09766,0.1367)	0.1299 (0.1143,0.1318)	0.09863 (0.08984,0.1096)
**2nd harmonic [g]**	0.1514 (0.1055,0.1855)	0.1816 (0.1328,0.2344)	0.1064 (0.1035,0.2461)	0.1226 (0.0957,0.1865)
**3rd harmonic [g]**	0.1816 (0.09473,0.251)	0.248 (0.1523,0.3379)	0.2197 (0.1152,0.3457)	0.1602 (0.09961,0.2002)
**4th harmonic [g]**	0.2129 (0.1455,0.3047)	0.29 (0.166,0.4863)	0.3125 (0.09766,0.3594)	0.1895 (0.1218,0.2661)
**5th harmonic [g]**	0.2197 (0.1416,0.3438)	0.3418 (0.1641,0.5508)	0.4551 (0.2041,0.4883)	0.189 (0.1367,0.2932)
**6th harmonic [g]**	0.2568 (0.1357,0.4746)	0.4316 (0.2734,0.7803)	1.695 (0.4854,1.841)	0.2617 (0.1765,0.405)
**7th harmonic [g]**	0.3262 (0.1758,0.4414)	0.5449 (0.3438,1.083)	1.665 (0.2529,1.933)	0.2886 (0.1748,0.439)
**8th harmonic [g]**	0.3125 (0.2402,0.8379)	0.7148 (0.4326,1.396)	0.9814 (0.8428,1.125)	0.2998 (0.188,0.4963)
**9th harmonic [g]**	0.3779 (0.1875,0.7461)	0.8271 (0.4775,1.445)	1.311 (0.459,1.521)	0.3506 (0.2354,0.6296)
**10th harmonic [g]**	0.5693 (0.3555,1.753)	1.255 (0.6025,1.84)	1.851 (0.5791,2.309)	0.3882 (0.2615,0.7036)
