# Supplementary figures and images for: Resolving the identification of weak‐flying insects during flight: a coupling between rigorous data processing and biology
Source: Agric For Entomol. 2021 Jun 2;23(4):489–505. doi: 10.1111/afe.12453 (PMC8596709; doi:10.1111/afe.12453)

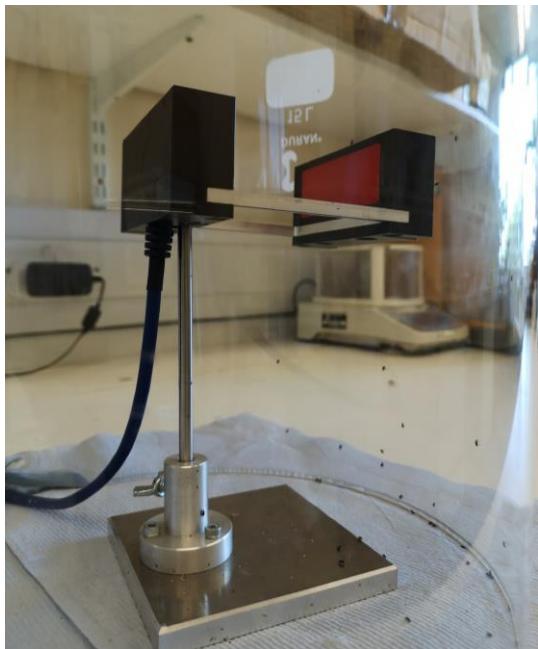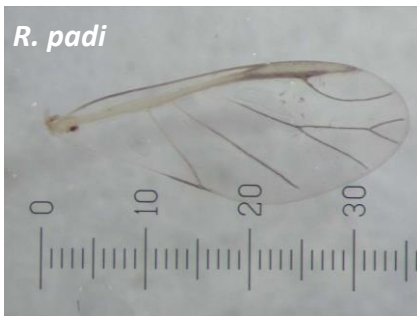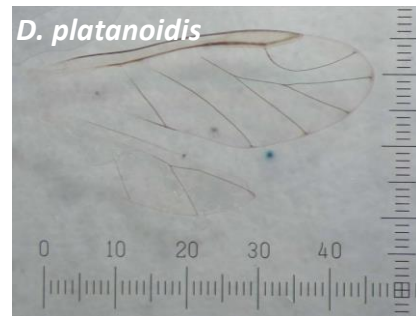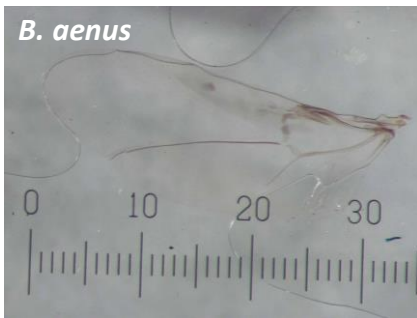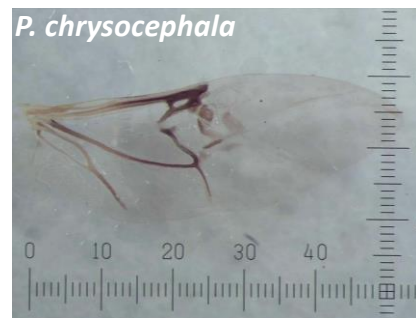

Supplement: Supplementary file 5 — Figure S1. Photograph of the experimental setup where the opto‐acoustic sensor is contained within a large jar allowing aphids to fly freely through the sensor. Photographs of illustrative wings from R. padi, D. platanoidis, B. aenus and P. chrysocephala. Each tick mark on the scale bar is 0.1 mm. [file AFE-23-489-s008.pdf]

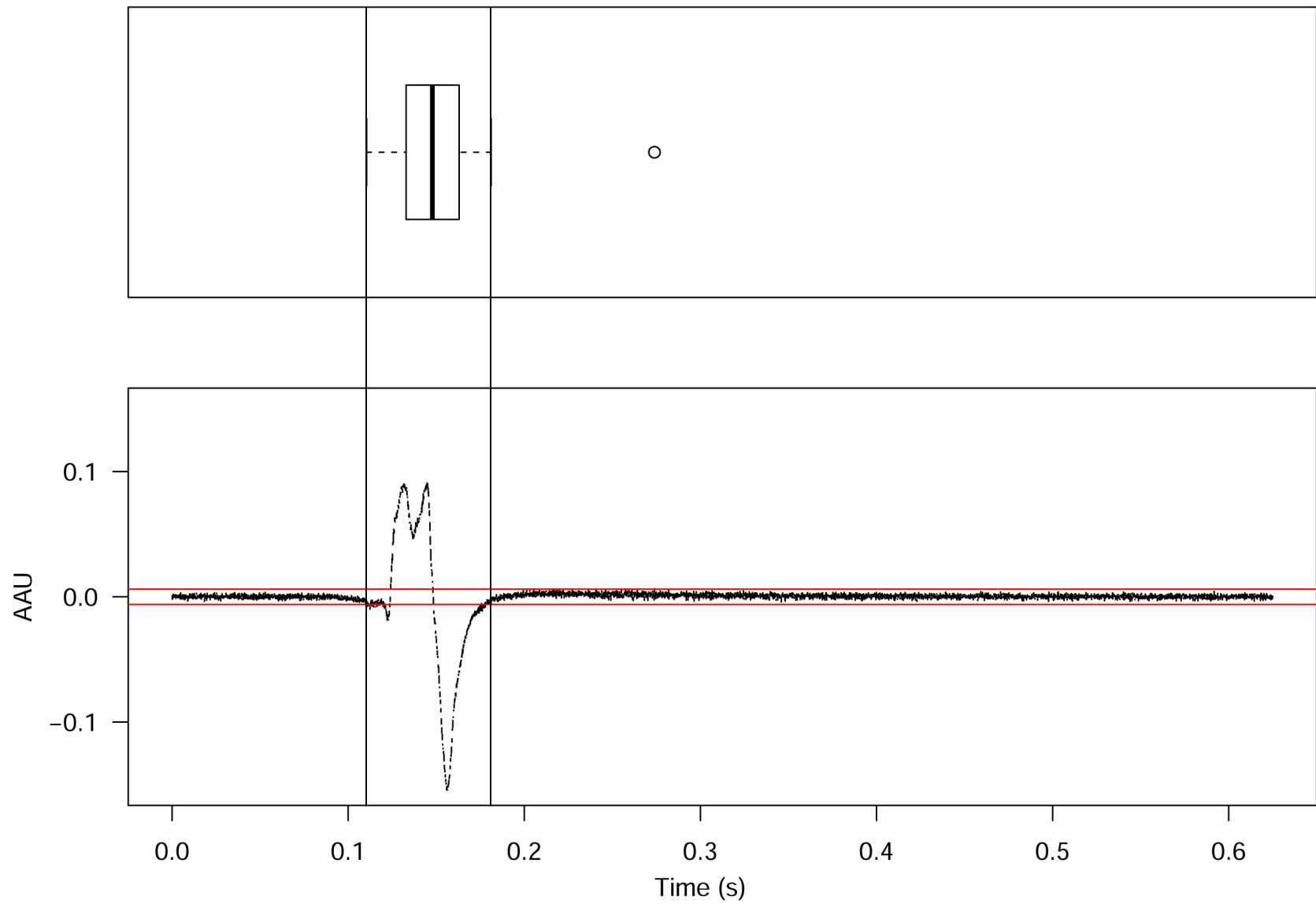

Supplement: Supplementary file 6 — Figure S2. Data processing. Figure illustrates how each recording is trimmed to remove periods of silence at the start or end of a recording. Lower panel is the audio recording, red lines are the threshold of ±0.0061 arbitrary amplitude units, above and below which sound is considered silence. The upper panel shows a box plot of the temporal indices exceeding this threshold. Audio is then trimmed to the whiskers of the boxplot defined as the largest (smallest) temporal index not exceeding three times the interquartile range away from the upper (lower) quartile, shown by the blue lines. [file AFE-23-489-s010.pdf]

a)

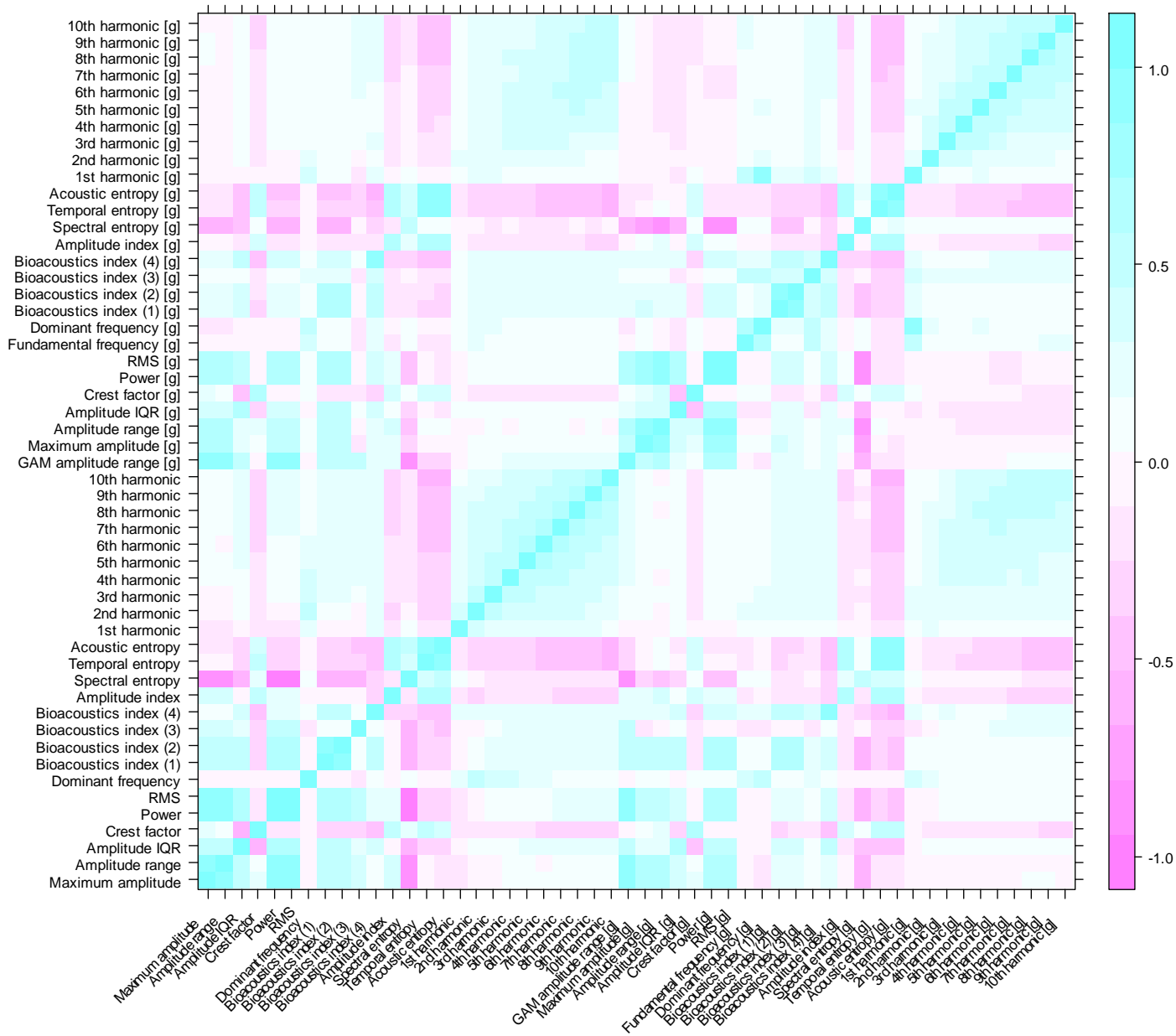

b)

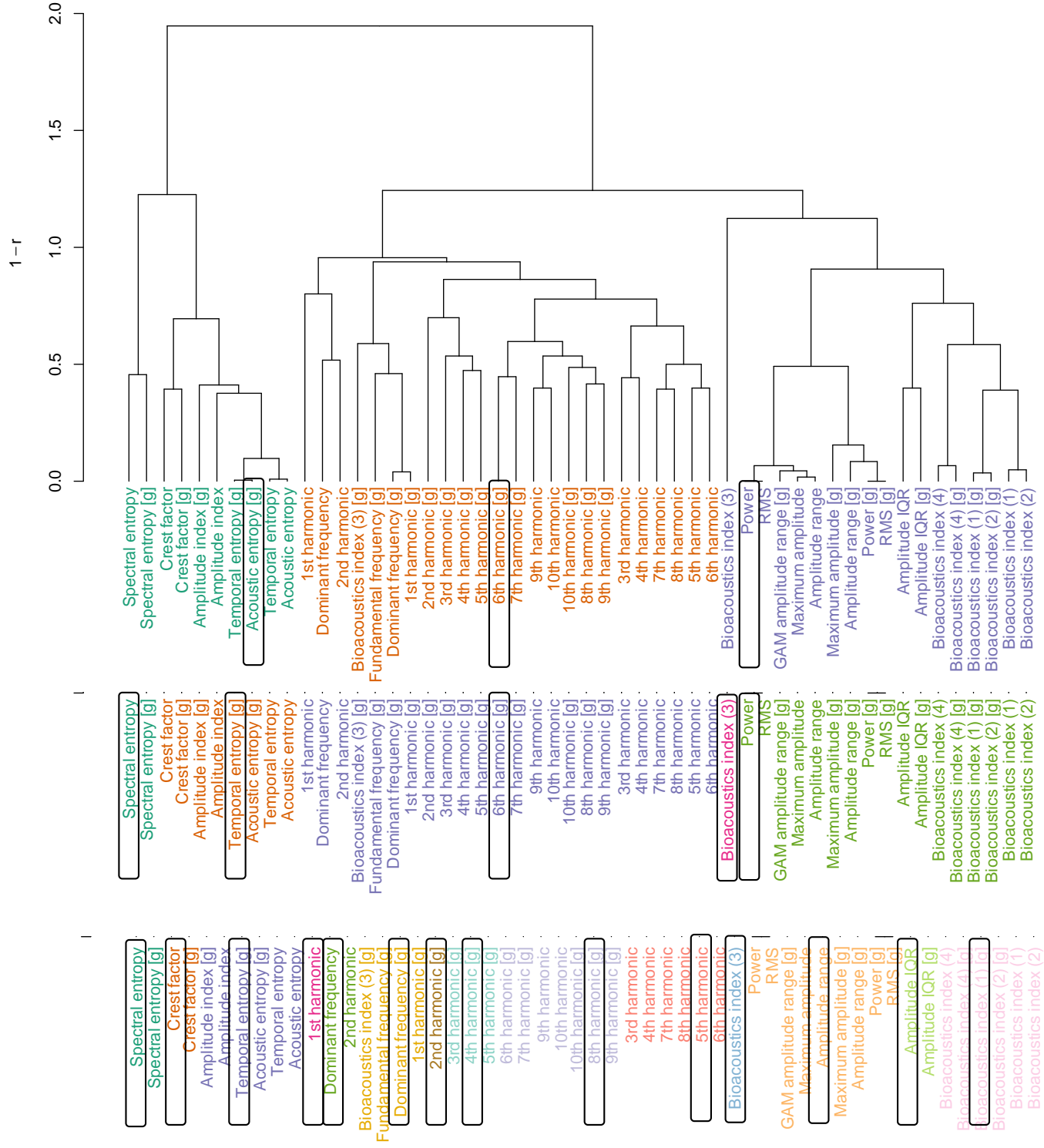

Supplement: Supplementary file 7 — Figure S3. Identification of a minimal feature set. (A) The correlation matrix between features calculated after standardisation. (B) Dendrogram of a hierarchical cluster analysis using complete linkage on 1 – r, where r is the correlation matrix of the standardised feature set. Features are coloured according to cutting the dendrogram into (i) 3 groups, (ii) 5 groups (iii) 14 groups. Features deemed most representative of each group are indicated by the box. [file AFE-23-489-s001.pdf]

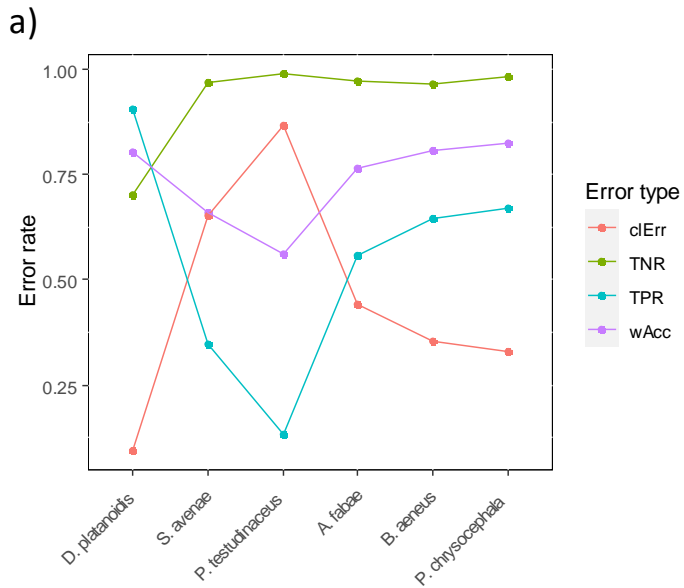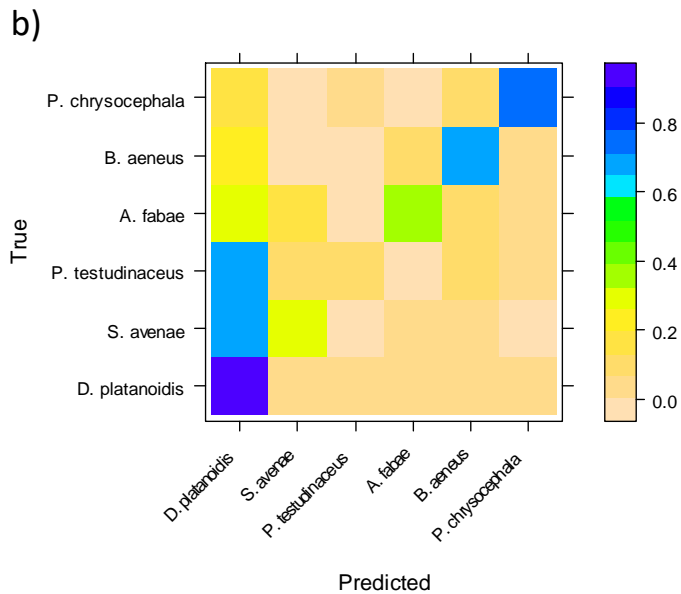

Supplement: Supplementary file 8 — Figure S4. Species classification error rates. (A) shows the class specific error rates where clErr is the class error rate (or 1 – true positive rate per class), TNR is the class specific true negative rate, TPR is the class specific true positive rate and wAcc is the weighted accuracy (wAcc = 0.5 × TNR + 0.5 × TPR). (B) shows the confusion matrices of classification predictions on the validation dataset, presented as a proportion per species. [file AFE-23-489-s006.pdf]

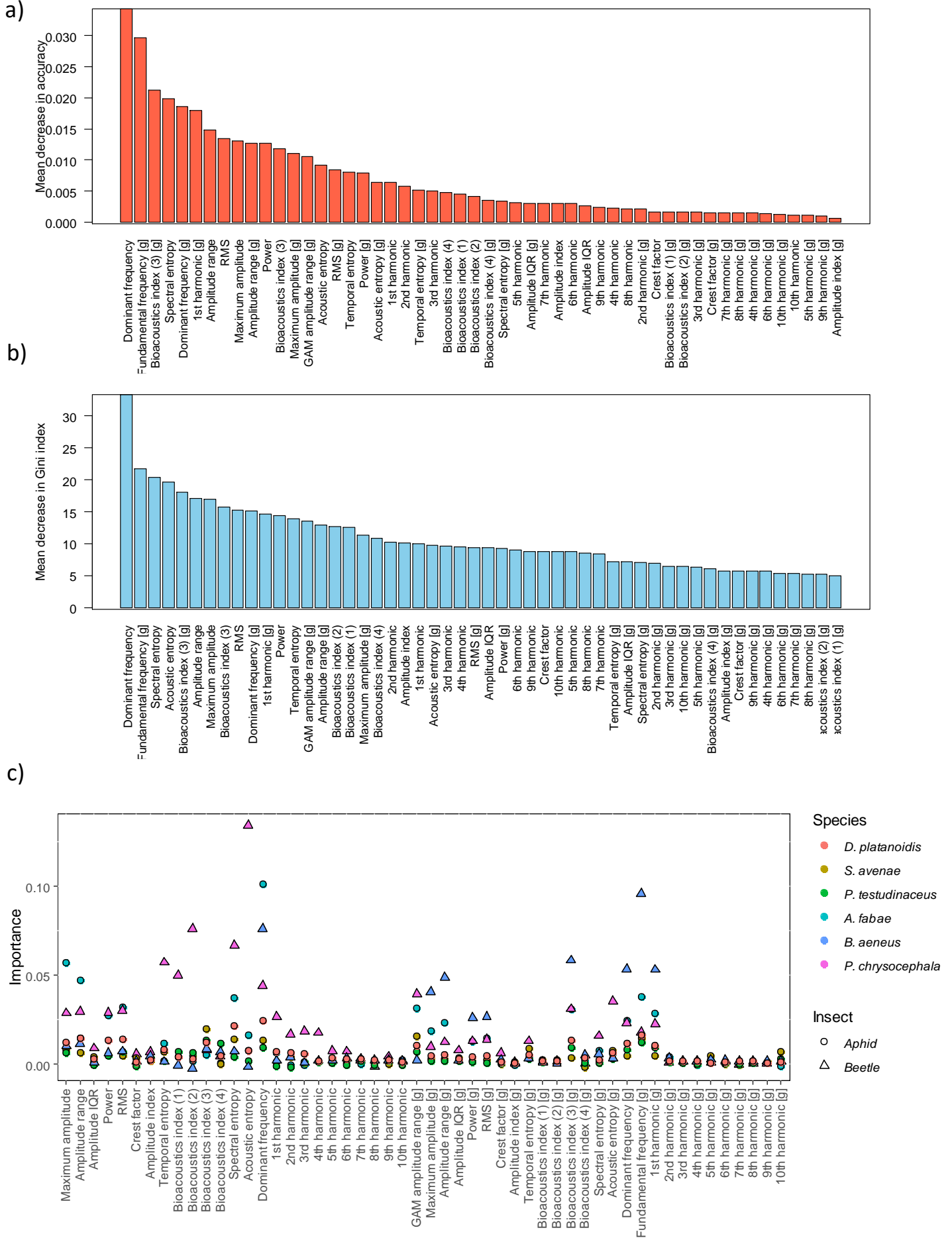

Supplement: Supplementary file 9 — Figure S5. Species classification. (A,B) give the mean decrease in accuracy and Gini index respectively for each feature variable considered in the model and (C) presents the within species importance of each feature variable. Feature variables denoted by (g) are derived after a detrending step (see Fig. 1) [file AFE-23-489-s002.pdf]

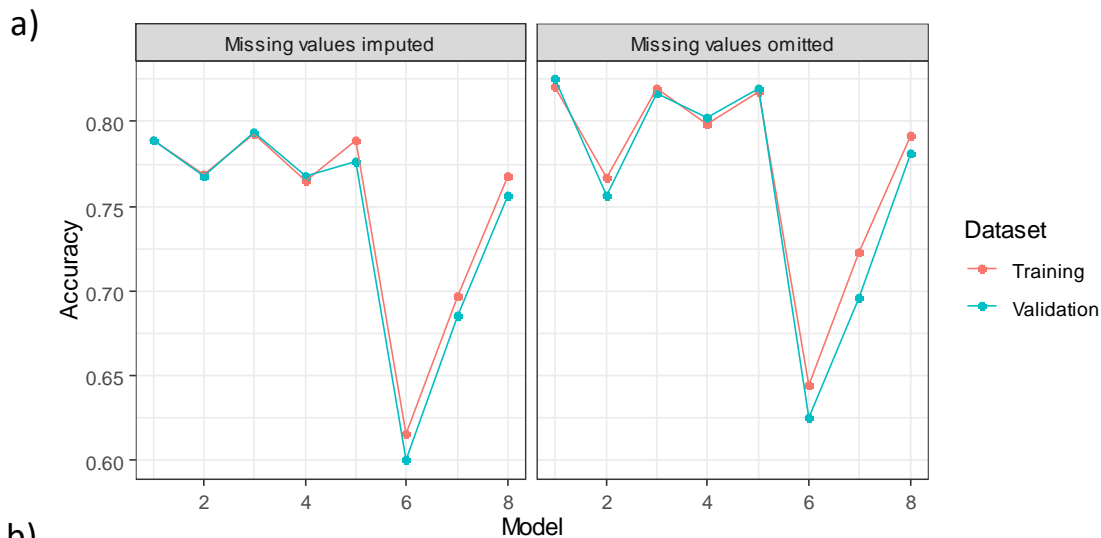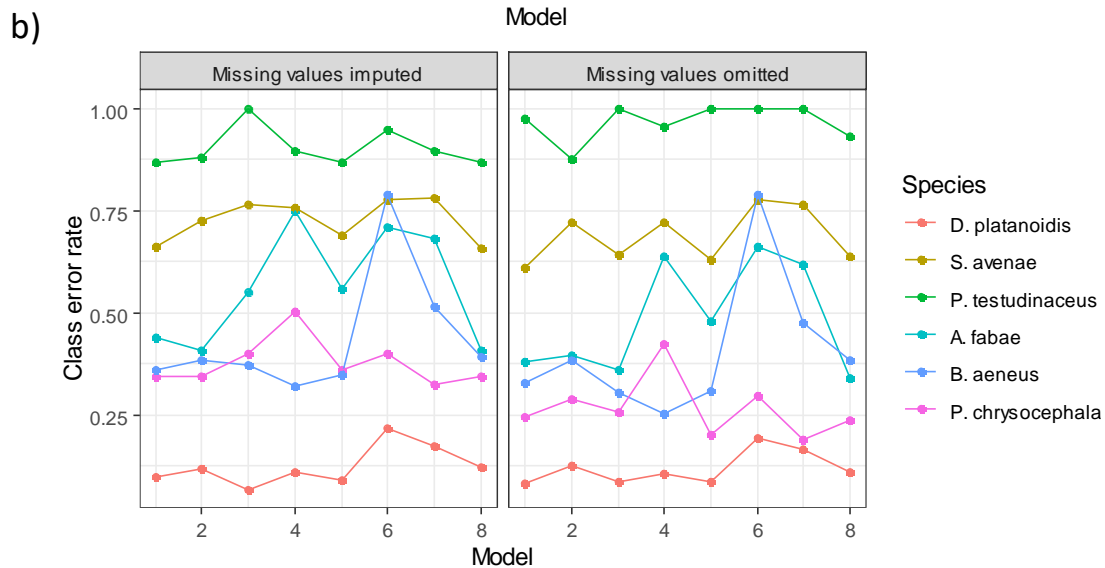

Supplement: Supplementary file 10 — Figure S6. Comparison of feature sets. (A) The mean accuracy rate on the out‐of‐bag predictions from the training set and on the validation set for both omitting and imputing observations with missing values. (B) the class specific error rates for each feature set for both omitting and imputing observations with missing values. Each model corresponds to a different subset of feature variables as detailed in Table 3. [file AFE-23-489-s009.pdf]
